# Forecasting the effectiveness of indoor residual spraying for reducing dengue burden

**DOI:** 10.1371/journal.pntd.0006570

**Published:** 2018-06-25

**Authors:** Thomas J. Hladish, Carl A. B. Pearson, Diana Patricia Rojas, Hector Gomez-Dantes, M. Elizabeth Halloran, Gonzalo M. Vazquez-Prokopec, Ira M. Longini

**Affiliations:** 1 Department of Biology, University of Florida, Gainesville, FL, USA; 2 Emerging Pathogens Institute, University of Florida, Gainesville, FL, USA; 3 Very Good Research & Development, LLC, Gainesville, FL, USA; 4 Department of Epidemiology, University of Florida, Gainesville, FL, USA; 5 Health Systems Research Center, National Institute of Public Health, Cuernavaca, Mexico; 6 Vaccine and Infectious Disease Division, Fred Hutchinson Cancer Research Center, Seattle, WA, USA; 7 Center for Inference and Dynamics of Infectious Diseases, Seattle, WA, USA; 8 Department of Biostatistics, University of Washington, Seattle, WA, USA; 9 Department of Environmental Sciences, Emory University, Atlanta, GA, USA; 10 Department of Biostatistics, University of Florida, Gainesville, FL, USA; University of Texas Medical Branch, UNITED STATES

## Abstract

**Background:**

Historically, mosquito control programs successfully helped contain malaria and yellow fever, but recent efforts have been unable to halt the spread of dengue, chikungunya, or Zika, all transmitted by *Aedes* mosquitoes. Using a dengue transmission model and results from indoor residual spraying (IRS) field experiments, we investigated how IRS-like campaign scenarios could effectively control dengue in an endemic setting.

**Methods and findings:**

In our model, we found that high levels of household coverage (75% treated once per year), applied proactively before the typical dengue season could reduce symptomatic infections by 89.7% (median of 1000 simulations; interquartile range [IQR]:[83.0%, 94.8%]) in year one and 78.2% (IQR: [71.2%, 88.0%]) cumulatively over the first five years of an annual program. Lower coverage had correspondingly lower effectiveness, as did reactive campaigns. Though less effective than preventative campaigns, reactive and even post-epidemic interventions retain some effectiveness; these campaigns disrupt inter-seasonal transmission, highlighting an off-season control opportunity. Regardless, none of the campaign scenarios maintain their initial effectiveness beyond two seasons, instead stabilizing at much lower levels of benefit: in year 20, median effectiveness was only 27.3% (IQR: [-21.3%, 56.6%]). Furthermore, simply ceasing an initially successful program exposes a population with lowered herd immunity to the same historical threat, and we observed outbreaks more than four-fold larger than pre-intervention outbreaks. These results do not take into account evolving insecticide resistance, thus long-term effectiveness may be lower if new, efficacious insecticides are not developed.

**Conclusions:**

Using a detailed agent-based dengue transmission model for Yucatán State, Mexico, we predict that high coverage indoor residual spraying (IRS) interventions can largely eliminate transmission for a few years, when applied a few months before the typical seasonal epidemic peak. However, vector control succeeds by preventing infections, which precludes natural immunization. Thus, as a population benefits from mosquito control, it gradually loses naturally acquired herd immunity, and the control effectiveness declines; this occurs across all of our modeled scenarios, and is consistent with other empirical work. Long term control that maintains early effectiveness would require some combination of increasing investment, complementary interventions such as vaccination, and control programs across a broad region to diminish risk of importation.

## Author summary

Using realistic simulation of dengue in the state of Yucatán, Mexico, we show high coverage indoor residual spraying (IRS) interventions can largely eliminate transmission for a few years, when applied proactively. However, initial success relies on population-level immunity, which declines with reduced infection rates, so simulated IRS campaigns stabilize at much lower effectiveness than initially observed. Moreover, if a campaign then suddenly stops, the model predicts large outbreaks until population immunity recovers. These results suggest that mosquito control could enable elimination in endemic settings, but that natural infections must be replaced, *e.g*. with vaccination, to achieve that end. Regardless, early campaign years’ performance cannot be assumed representative of longterm benefit, and campaign cost estimates must account for increasing population susceptibility.

## Introduction

Dengue is a mosquito-borne viral pathogen resulting in an estimated annual global economic burden of 9 billion USD [1]. Dengue incidence has trended upward the last several decades due to a combination of increased urbanization, human mobility and trade, irregular water supplies, climatological changes, and ineffective or unsustainable vector control [2]. The complex human immune response to dengue—particularly the temporary cross-protection between the four known serotypes, which fades and potentially enhances disease-risk—complicates public health responses [3]. Vaccine development has focused on creating vaccines efficacious against all four serotypes, and several such vaccines are currently in clinical trials [4]. Sanofi-Pasteur produced a vaccine (known as CYD-TDV or Dengvaxia) that has been licensed in several countries, but trial results indicate that its efficacy depends on past dengue exposure [5, 6], leading some to hypothesize that the vaccine may act like a natural infection, providing temporary protection but increasing the disease risk for recipients with no infection history [7]. Based on these results, the Sanofi-Pasteur vaccine may not in practice be effective in regions with low or moderate seroprevalence [8, 9]. As more trial data has become available, Sanofi-Pasteur has advised national health organizations and medical professionals to only vaccinate patients with likely prior dengue infection [10].

Until a cost-effective, broadly efficacious vaccine becomes available, the principle dengue intervention will remain vector control. New technologies, such as releasing Wolbachia-infected or genetically modified mosquitoes, sterilizing techniques, and mass-trapping are at different stages of development [11]. Practical deployment of these may take many years, while spread of dengue and other *Aedes*-borne diseases continue unimpeded. Hence, research evaluating the effectiveness of existing vector control approaches is a pressing endeavor [12, 13].

Existing control activities have focused on the primary vectors, *Ae. aegypti* and *Ae. albopictus* [14], with approaches directed against both immature aquatic stages and adult mosquitoes [15, 16]. History indicates that vector-control approaches can work—the near-elimination of *Ae. aegypti* from Latin America is a well-documented example of thorough, well-funded, and successful vector control [17]—however, recent efforts have failed to prevent the resurgence and expansion of *Ae. aegypti* and *Ae. albopictus* [18] and subsequent regional expansion and intensification of dengue, chikungunya, and Zika. Cost is certainly a factor. Vector control using insecticides (*e.g*. space-spraying, larvicides) requires skilled staff and stable funding, and even then some approaches may be ineffective for controlling dengue [19]. Some approaches, like mitigating potential breeding sites, only work with widespread community participation [12, 20], and are not sustainable as implemented in many locations [15, 21]. Modern vector control activities are not sufficient to control dengue, and because epidemiological assessments are typically neglected, we lack data on the potential effectiveness of these activities on dengue or any of the other arborviruses [15, 21]. While new and old vector control interventions have some promise, the data challenges prevent public health officials from estimating how successful interventions are likely to be, and what options they have to ensure effective control efforts.

Indoor residual spraying (IRS) is an example of a well-understood approach with potentially under-exploited promise for *Ae. aegypti* control. IRS is the treatment of common mosquito resting surfaces inside houses with a long-lasting insecticide. Because *Ae. aegypti* strongly prefer feeding on humans and rest primarily indoors, they are more likely to be reached by IRS than by space sprays, especially outdoor treatment [22]. Like other insecticide-based approaches, IRS necessitates specialized training, is time-consuming to deliver, may require a campaign to garner public acceptance, and must be tailored to region-specific factors, such as the insecticide resistance of local mosquitoes. Before making these investments, public health officials will likely require information on optimal deployment, scalability, and the long-term effectiveness of IRS. Large scale empirical studies to answer these questions are costly and take many years, but mathematical models–calibrated by real epidemic data and parametrized based on small-scale empirical studies on IRS entomological efficacy (*i.e*., effect on mosquito mortality) and durability–can provide a reasonable basis for action.

IRS appears to have a large impact on *Ae. aegypti* mortality. IRS campaigns to eradicate malaria in the Mediterranean region appear to have led to the elimination of *Ae. aegypti* [23], and IRS either alone [24], or in combination with larval control [25], contributed to the elimination of *Ae. aegypti* from Guyana and the Cayman islands, respectively. Work in Iquitos, Peru, indicates that treating household surfaces with IRS reduces the likelihood of observing adult mosquitoes by >70% up to 12 weeks post spraying [26], and experimental research in Cairns, Australia indicates IRS led to 86-96% reduction in dengue cases in sprayed premises, compared to unsprayed controls [27]. All of these results indicate high IRS efficacy. Regarding durability, current IRS insecticides last an estimated three months, but new formulations might last as long as five to eight months [28].

Regarding the potential evolution of insecticide resistance in the vector, recent work in Mérida, Mexico, showed that high resistance to pyrethroids in *Ae. aegypti* can be controlled when implementing IRS using alternative formulations, such as bendiocarb [29]. Such results, combined with the recent development of new IRS formulations [30, 31], suggest a practical insecticide management plan for IRS where various formulations are rotated to counter the evolution of resistance [32] in order to maintain efficacy from year to year.

Based on these IRS efficacy and durability data, we present a framework for assessing IRS interventions. We integrated IRS into an existing stochastic simulation model [33] representing dengue transmission and burden in the Mexican state of Yucatán, and investigated the impact across several campaign options, including different household coverage levels, rollout periods, optimal seasonal timing of campaigns, and insecticide durability and efficacy.

## Materials and methods

The model is an agent-based, stochastic simulation of dengue transmission in the Mexican state of Yucatán; the model represents the state-wide population (approximately 1.82 million people) in households, with daily movement to workplaces and schools. Each location has a local mosquito population, infectious mosquitoes may migrate between adjacent locations, and all mosquito populations are modulated by seasonal forces (see Fig A in S1 Text). General details of the model remain the same as in [33], with detailed updates discussed in the SI, namely updated empirical data (Section 1 in S1 Text), more realistic human aging (Section 2.1 in S1 Text), serotype introduction (Section 2.2 in S1 Text), and mosquito age-at-infection and lifespan models (Sections 2.4, 2.5 in S1 Text), and refined fitting procedure (Section 3 in S1 Text). For all analyses, we sampled across the 1000 best parameter combinations from the fitting results. We match each intervention result to a baseline non-intervention result using the same parameter combination. We report median values from the sampled results.

To fit the model, we simulate a long burn-in period to establish a stable population-level immunity distribution, followed by a fitting period with known serotype introductions. Model output (dengue cases and seroprevalence) from the fitting period is compared to empirical data. For forecasting, we use the same periods as fitting, then switch to introducing all serotypes for another stabilization period, and finally introduce the interventions and repeat them annually for the next 40 simulated years. The first 10 years are emphasized in the main text, and the entire forecast period is reported in the SI; see Fig F-H in S1 Text.

In a given intervention scenario, there are five IRS campaign properties: the campaign start date, coverage level, IRS durability, IRS efficacy, and rollout period. Campaign start date refers to the first day of the year that any house will be treated with IRS. We considered start dates spaced one week apart (January 1, January 8, …) for effectiveness sensitivity studies, and the “proactive” (late May) and “reactive” (mid November) approximate extrema for longer term analyses. Coverage level determines the proportion of households that will be selected for IRS treatment in a given year; we considered 25, 50, and 75% coverage levels. Durability is how long the IRS effect lasts after treating a house; we considered durabilities of 30, 90, and 150 days. Efficacy is how much IRS reduces the mosquito population at that location; we considered efficacies of 40, 60, and 80%. Rollout period determines how many days it takes to treat all the selected houses; we considered 1 day (all houses covered in a single day), 90 days, and 365 days (selected houses treated randomly throughout the year). Houses experience the IRS effect if their last treatment is within the IRS durability window. See Section 2.7 and Fig A-C in S1 Text for campaign model details.

We select treated households randomly throughout the entire model population; real mosquito control campaigns would no doubt have particular spatial distribution, but we make the modeling simplification that they are random. IRS is not applied to workplaces or schools, so transmission outside households is not affected. We model IRS in treated households as (1) reducing the location’s susceptible mosquito population and (2) increasing daily mortality probability for infectious mosquitoes. For (1) we use the scenario IRS efficacy to reduce the susceptible population (*e.g*., by 80% to 20% of the original value), and for (2) we calculate a daily additional mortality probability associated with the overall population reduction (*e.g*., roughly 0.13 for 80% efficacy IRS). This additional mortality is treated separately from the normal daily mortality due to age or other factors. To calculate daily mortality, we use the assumed IRS efficacy combined with our empirically-derived model for mosquito age distribution. The age distribution determines baseline daily mortalities, to which we add a constant daily death probability; see Section 2.6 in S1 Text for details.

We compile the model using GCC [34], importing GSL [35] for pseudo-random number generation, and perform simulations on the University of Florida High Performance Computing Cluster. On an AMD Opteron 4284, each model run takes ~2 minutes per simulated year for the Yucatán population (1.82 million). Memory use is ~0.7 GB for Yucatán. The C++ source code is available at https://github.com/tjhladish/dengue. We use R for analysis of simulated data and figure generation [36]. We expand our previous model fit to include more long-term clinical and severe case data (Table A in S1 Text) [37] and a 1987 serosurvey [38, 39]; we also use an updated AbcSmc [40], which now includes covariance when selecting new parameter combinations.

## Results

**Fig 1:**
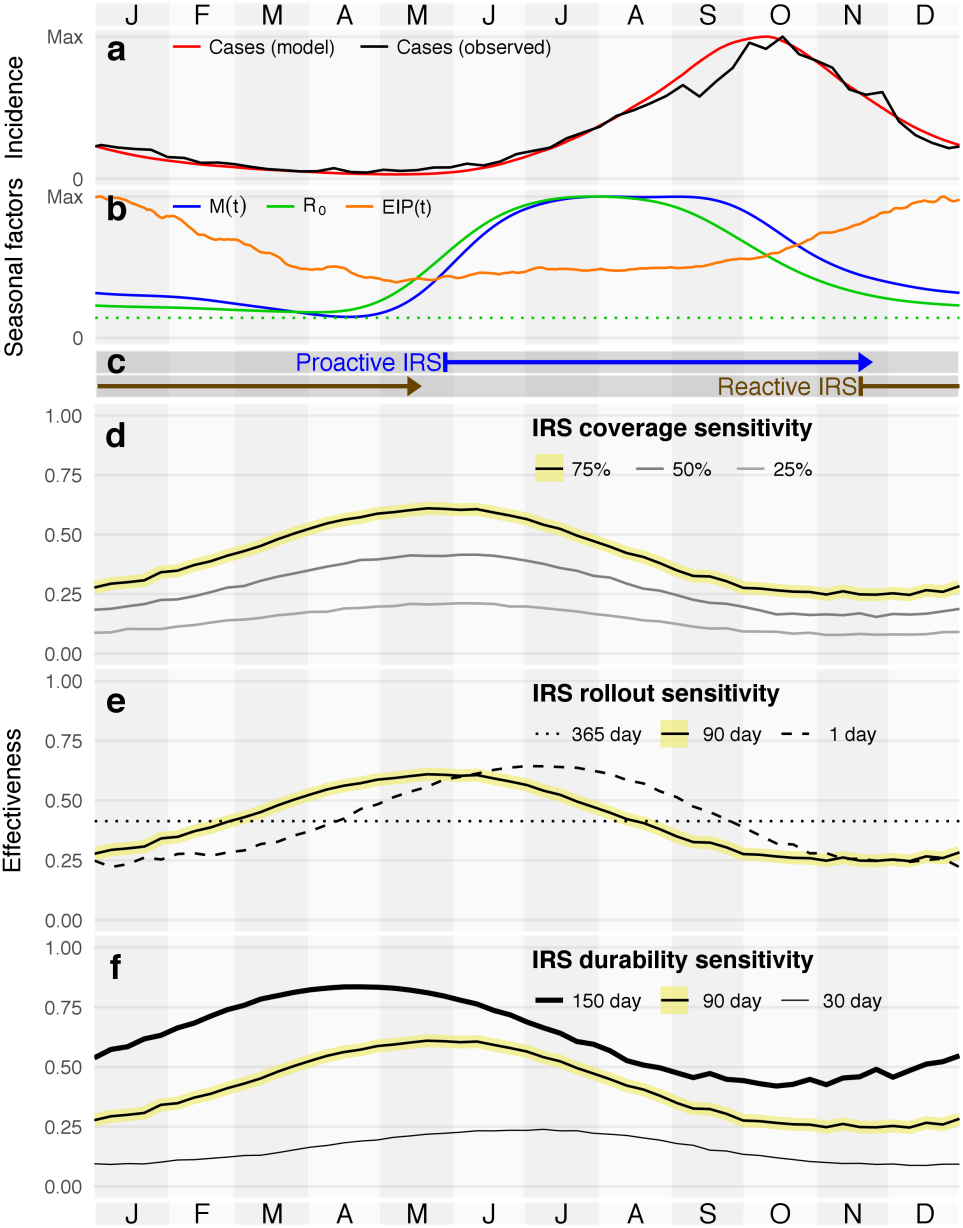
Seasonality of dengue in the state of Yucatán (a-b), reference campaign periods (c), and effect of campaign start date on IRS cumulative 10 year effectiveness, in combination with three additional sensitivity dimensions (d-f; see Fig K, panel g in S1 Text for efficacy sensitivity). Effectiveness sensitivity plots have the reference scenario highlighted in yellow. (a) Observed and modeled average dengue cases in the state of Yucatán, 1995-2015. (b) Model seasonal components: extrinsic incubation period (EIP) and mosquito population size (M(t)) combine to drive basic reproductive number (*R*_0_); *R*_0_ = 1 (the epidemic threshold in a fully susceptible population, dotted green) provided for reference. (c) Reference insecticide-active periods for 90 day rollout campaigns with 90 days of IRS durability, from start (treatment of first house, left bar) to end (insecticide expiration from last house, arrowhead). (d-f) Sensitivity to IRS coverage, with 90-day campaigns, 90-day durability, 80% efficacy, and 75% coverage as the reference scenario. Each sub-plot shows a univariate sensitivity study of ten-year effectiveness by start date from that reference.

Using our dengue model representing the state of Yucatán, Mexico, we assessed several options for IRS-like interventions. The strong seasonality of dengue incidence in Yucatán and many other endemic areas suggests that annual timing of transient interventions may be important. The model reproduces, but is not fitted to, observed dengue seasonality, using temperature and precipitation data for Mérida, the largest city in and state capital of Yucatán (Fig 1a and 1b).

We report intervention impact by comparing model runs with and without interventions. We focus primarily on effectiveness, *IRS*_eff,Δ*t*_, which we calculate from incidence of symptomatic infections (*i.e*., cases) in the baseline and intervention model runs as

$$\begin{array}{c} \begin{array}{c} IRS_{\text{eff},\Delta t}=1-\frac{\text{cases}\;\text{with}\;\text{IRS}\;\text{intervention}}{\text{cases}\;\text{without}\;\text{intervention}} \end{array} \end{array}$$

for a particular time period, Δ*t*, in years. We primarily consider two reporting periods: the first ten years, cumulatively (*IRS*_eff,10_), that the intervention is active, or for a year at a time in a series of years (*IRS*_eff,1_). For both baseline and intervention scenarios, the model simulates many years of dengue dynamics to normalize population immunity before interventions are considered; see Materials and methods and Section 2.8 in S1 Text for details.

We focused on first determining the optimal timing of IRS deployment, and then used that optimal timing to consider more detailed assessment of IRS effectiveness for the reference campaign; Fig 1c shows campaign periods we used for these additional analyses.

### Seasonality of intervention effectiveness & optimal timing

We considered different annual campaign start dates for several sensitivity dimensions (Fig 1d–1f) and calculated the cumulative effectiveness over the first 10 years of active intervention, *IRS*_eff,10_. For sensitivity analyses, we considered five features: start date (x-axis in each panel), campaign coverage (Fig 1d), campaign rollout period (Fig 1e), IRS durability (Fig 1f), and IRS efficacy (Fig K, panel g in S1 Text). The efficacy response is qualitatively similar to the response to changes in coverage. We maintained a consistent reference scenario for each study, a 90 day campaign at 75% coverage with 90 day IRS durability and 80% IRS efficacy, and for each analysis only changed one of the features from that reference. For each feature, we considered three levels. Table 1 reports the effectiveness maxima, minima, and associated dates.

**Table 1:** High and low 10-year median effectiveness for sensitivity analyses, with the reference scenario highlighted as in Fig 1d–1f. Date is the campaign start day, which is irrelevant for continuous (365 day rollout) campaigns.

Coverage (%)	Maxima		Minima		$\frac{\text{Max}-\text{Min}}{\text{Min}}$
	Date	*IRS*_eff,10_	Date	*IRS*_eff,10_	
75	21 May	0.61	10 Dec	0.25	1.47
50	11 Jun	0.42	26 Nov	0.15	1.69
25	04 Jun	0.21	05 Nov	0.08	1.70
Rollout (days)					
1	02 Jul	0.64	31 Dec	0.22	1.91
90	21 May	0.61	10 Dec	0.25	1.47
365	-	0.41	-	0.41	-
Durability (days)					
150	23 Apr	0.84	15 Oct	0.42	0.99
90	21 May	0.61	10 Dec	0.25	1.47
30	09 Jul	0.24	17 Dec	0.09	1.71
Efficacy (%)					
80	21 May	0.61	10 Dec	0.25	1.47
60	21 May	0.43	12 Nov	0.17	1.58
40	28 May	0.27	05 Nov	0.11	1.42

Increasing IRS coverage (*i.e*., treating additional houses) increases the maximum 10-year cumulative effectiveness, as does increasing delivery speed (*i.e*., decreasing the number of days to rollout the intervention) and increasing IRS durability (*i.e*., how long IRS is active at a treated location after application) and efficacy (*i.e*., how much the IRS decreases the local mosquito population). Increasing coverage, durability, and efficacy also increase minimum effectiveness. In those cases, the maximum increases more than minimum, meaning the absolute gain by deploying optimally is also higher. However, by examining the ratio of max and min, we observe a pattern of diminishing returns for both coverage and durability, but not for efficacy. Rollout speed has a different effect: in addition to more precisely targeting the best day for treatment, it appears also possible to more precisely target the worst day.

Changes to rollout periods had a less pronounced effect, with shorter, more intensive campaigns achieving better peak effectiveness. Optimally-timed 1-day campaigns slightly out-perform optimally-timed 90-day campaigns, and both out-perform continuous campaigns. Continuous campaigns, however, outperform the shorter campaigns when they are poorly timed. Although we focus on results for 75% coverage here, results were qualitatively identical for 50% and 25% coverage; see Fig D in S1 Text for details.

Changing either campaign rollout period or IRS durability shifts the optimal start date. For example, the 1-day rollout curve lags roughly 45 days after the 90-day curve, or approximately half of the treatment window. However, if we compare campaigns’ “insecticide active” windows (the sum of the campaign rollout period and IRS durability) instead of start date, all optimal interventions share the same midpoint (late August to early September; see Fig D,E in S1 Text). This trend means that all optimal campaign options build coverage while *R*_0_ is at its high plateau, and then coverage decays with *R*_0_.

### Proactive and reactive campaign start dates

The maximal and minimal effectiveness respectively correspond to campaigns that are proactive—*i.e*., applied when transmission first becomes likely, but before many cases are observed—and reactive—*i.e*., applied in response to peak cases, when other factors are already driving down transmission probability. In the real world, there would likely be a public health organization response to dengue-related observations (*e.g*., a certain number of cases), triggered at different times from year-to-year. Since seasonal drivers in our model do not vary year-to-year, however, we approximate these proactive and reactive responses with fixed dates. We use May 27 (proactive) and November 18 (reactive) as general approximations of the best and worst timing, respectively, for campaigns with 90-day rollouts and 90-day durabilities; see Fig 1c and 1d. Proactive campaigns directly reduce transmission by substantially curtailing what would otherwise be large populations of mosquitoes (due to increased precipitation) that have shorter incubation periods (due to warmer weather). As reactive campaigns tend to start after epidemics have peaked, they have little direct impact during epidemics; instead, they increase the likelihood of extinction for off-season transmission chains (Fig I in S1 Text). Fewer off-season transmission chains means outbreaks in subsequent years must be initiated by random introductions, rather than beginning immediately from chains that have survived the off-season. Although proactive campaigns are better, the delay in epidemic onset achieved by reactive campaigns results in substantial effectiveness, despite there being no control during most of the epidemic. Our more detailed model agrees with previous work that used more homogeneous assumptions (*e.g*., without age structure or explicit households) on space-spraying-based dengue control [41], as well as IRS-based control of malaria [42].

### Long-term annual effectiveness

**Fig 2:**
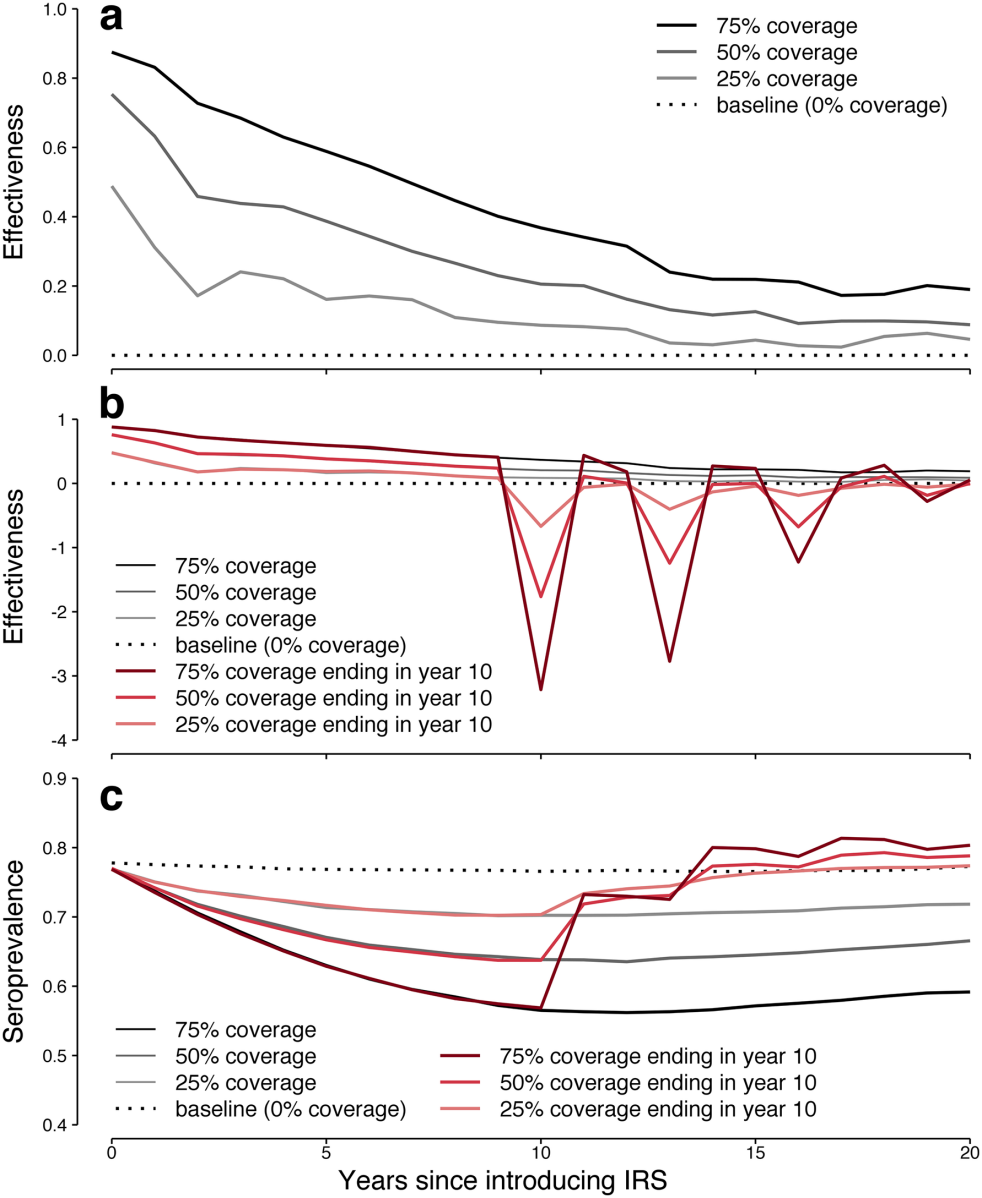
Predicted overall effectiveness of IRS and population immunity over 20 years. (a) New IRS campaigns show initial high effectiveness that wanes over time even without the evolution of insecticide resistance in mosquitoes. (b) If campaigns that have been effective are abruptly stopped, or if, for example, mosquitoes were to suddenly evolve complete insecticide resistance (red), epidemics much larger than baseline would likely occur until the human population re-established a high level of immunity. (c) At baseline (dashed black), a consistent fraction of the population is expected to have some level of naturally acquired immunity (seroprevalence; see Fig H in S1 Text for detailed breakdown). Because IRS is effective in reducing dengue infections (solid black, grey), seroprevalence decreases over time, permitting somewhat larger epidemics, but still smaller than baseline. If IRS is stopped or abruptly loses efficacy (red), population immunity rapidly climbs in response to the resulting very large epidemics.

Effectiveness wanes over time, even for optimal proactive timing, as naturally acquired immunity declines (see Fig 2a). Initially, the 75% coverage, proactive intervention prevents nearly 90% of cases (0.87 effectiveness in the first year). However, within 10 years, this benefit is cut in half, and to less than a quarter by 20 years, as the population seroprevalence also declines (see Fig 2c, black lines). Effectiveness continues to dip further (see Fig G in S1 Text for the time series out to 40 years), though it eventually rebounds to settle around 0.23 (roughly a quarter of the original value). A similar pattern emerges for the other coverage levels, though shifted earlier and more pronounced: 50% coverage drops from its initial 0.75 effectiveness to less than a quarter of that before year 15, ultimately settling around 0.14 (a fifth of its initial value), and 25% coverage drops from initial 0.49 to less than a quarter of that before year 10, and ultimately settles around 0.07 (a seventh its initial value). All coverage levels see a dip in seroprevalence, corresponding to both the peak population benefit (in prevented cases) and risk (due to lack of immunizing infections). Seroprevalence ultimately rises as effectiveness declines, though not back to the initial levels, and this is what leads to the later observed increase in effectiveness.

### Discontinuing vector control

Although a successful vector control program is unlikely to be abruptly ended, in our model annual IRS effectiveness dropped by more than 50% by year 10 for all scenarios. It is plausible than an intervention deemed ineffective would be halted in favor of some alternative, or that efforts would be disrupted due to *e.g*., funding issues, conflict, or natural disasters. Alternatively, mosquitoes might cease to be killed for some other reason: IRS might be continued, but if mosquitoes abruptly evolved complete insecticide resistance, the epidemiological effect would be the same. We investigated the consequences of ending IRS after most of the initial effectiveness had waned.

Epidemic projections for IRS that is ended abruptly after 10 years are dramatic (Fig 2b). The more effective the modeled intervention had been, the larger the epidemics are in the first year without IRS. For previous 75% coverage, the median effectiveness of stopping IRS is −3.2, corresponding to epidemics that are 4.2 times larger than observed prior to initiating IRS. For lower coverage levels, since fewer natural infections are prevented, this swing is not as drastic, but still results in larger-than-pre-intervention epidemics for both 50% coverage (2.8x) and 25% coverage (1.7x) levels. In the long term, population seroprevalence, and thus epidemics, return to the non-intervention baselines. The sharp 3-year oscillatory behavior observed here is likely due to our model’s simplifying assumption that cross-serotype immunity lasts exactly two years, and would likely be less pronounced but still observable. After ten years without IRS, epidemic dynamics are approximately back to what they were pre-IRS.

### Mosquito population sensitivity

To investigate the applicability of these results to settings with other levels of dengue burden, we considered larger and smaller baseline mosquito populations (*M*_*peak*_); see Table 2 for summary results. Changing the mosquito population varies the exposure rate that people in the model experience (also known as the force of infection). Several other parameters contribute to the exposure rate, but of the fitted parameters, the mosquito population has the most direct effect on exposure rate.

**Table 2:** Effect of mosquito population on median cases averted and cumulative effectiveness of IRS. We considered both increasing and decreasing the number of mosquitoes in the model by 30% from the fitted population size.

Years	Cases averted per 100k		Overall cumulative effectiveness	
	1-5	6-10	1-5	6-10
70% *M*_*peak*_	4490	3647	0.82	0.68
Baseline *M*_*peak*_	4990	3409	0.74	0.49
130% *M*_*peak*_	4940	2901	0.67	0.37

For smaller *M*_*peak*_, there are generally fewer baseline cases, and thus fewer cases to prevent. However, a higher fraction of those cases are prevented, so we see higher effectiveness. This is the expected non-linear dynamic for systems approaching the epidemic threshold. The same non-linear effect explains results for higher *M*_*peak*_: well above the epidemic threshold, there is much lower benefit for the same scale of intervention.

Regardless of mosquito population size, overall effects remain the same: effectiveness still wanes over time, and proactive timing still outperforms reactive timing (Fig J in S1 Text). For a sufficiently small mosquito population, we would expect to see durable eradication, but we did not consider an *M*_*peak*_ that low in our sensitivity studies.

## Discussion

Our model results indicate that an IRS-like approach that increases mosquito mortality in households can be effective at preventing dengue cases. Exact benefits will vary with local ecological and epidemiological factors, but we expect two main qualitative outcomes will apply when any efficacious, semi-durable vector control like IRS is introduced to an endemic region. First, proactive interventions will typically outperform reactive interventions. Second, these interventions will have effectiveness that wanes over a period of years (as observed in Singapore [43]) similar to the “honeymoon” effect predicted in some vaccine scenarios (*e.g*., measles [44]), and may be dangerous (as predicted for other vector control interventions [45]) to stop abruptly. We believe these conclusions would be broadly applicable in any discussion about IRS strategy, and that our modeling approach could be tuned to other locations and provide useful estimates of expected performance.

For our model of the state of Yucatán, we observed that IRS campaigns had the best performance when dengue incidence is still low, but transmission probability just started to increase. As additional houses are treated during the campaign, coverage increases, and as insecticide efficacy expires, coverage decreases. In general, we observed that the best-performing campaigns were timed so that maximum coverage coincided with the typical peak epidemic growth rate, occurring around September 1 in our model. To achieve this, we estimate the optimal start date should lead the peak in epidemic growth by the average of insecticide durability and campaign rollout period ((90+ 90)/2 = 90 days for the reference campaign). Timing appears to be insensitive to household coverage levels as well as the IRS efficacy. The general conclusion is consistent with previous work [41, 42], though we are able to produce more usable guidance by modeling multiple serotypes, a spatially explicit population, and more realistic mosquito seasonality and intervention deployments. Public health organizations considering IRS interventions should determine the historical timing of peak epidemic growth rate—not to be confused with the peak in cases—and start campaigns early enough that peak insecticide coverage occurs at the same time.

Highly effective campaigns need not be precisely timed, however. Our model indicates a month-long period (spanning late May) with little difference in effectiveness for starting campaigns. Practically, it will be difficult to accurately forecast the peak growth date as far ahead as a campaign should be starting in any given year. Accounting for detailed factors (*e.g*., current-year ecological, entomological, and epidemiological data) could inform campaign timing, but it may be more practicable to use historical averages to anticipate peak growth as we describe here. For regions with limited historical data, continuous campaigns (a more modest number of houses treated every day of the year) remain a viable option that still outperforms reacting to a recent epidemic.

Campaigns that do start reactively, when epidemics are typically waning, provide reduced, but still remarkable, effectiveness. That mosquito control could be effective at all when mosquito populations are already very low seems counter-intuitive. However, we found that with no intervention, our model exhibits a small number of inter-seasonal transmission chains (Fig I in S1 Text). At the end of a normal (non-intervention) epidemic season, enough infections remain that some are likely to find a series of susceptible hosts and persist until seasonal conditions again become favorable for high-level transmission. When that occurs, the epidemic can begin immediately, rather than having to wait for a random introduction. For regions with less off-season transmission than in our model, or a higher rate of external introductions, effectiveness of off-season interventions would be reduced further.

Even given optimal timing, we observed declining effectiveness for all campaign scenarios. The highest coverage levels were initially very promising, preventing over 80% of cases in years 1 and 2, but ultimately the effectiveness of all scenarios waned to less than 1/4 of their initially observed benefit, with lower coverage levels losing relatively more effectiveness. The reason for this change is similar to what drives the “honeymoon” effect in vaccine interventions: averted infections mean the natural immunization rate is lower, gradually decreasing population-level immunity. In the case of vector control, however, susceptibles accumulate more rapidly (via births) since natural immunizations are not being replaced. Our model assumes dengue infection causes life-long immunity to the infecting serotype [46], and 2 years of immunity to all other serotypes [47, 48]. Thus, in the early years of a new vector control strategy, the population benefits from both less exposure due to fewer mosquitoes, and a high level of immunity due to high exposure in recent history. As population immunity wanes, the same intervention, with no loss of efficacy (*i.e*., ability to kill mosquitoes), appears less effective. Of course, waning efficacy is an additional concern, as mosquito populations have been observed to evolve resistance to various insecticides. This is a key difference from the vaccine intervention honeymoon effect, since there are multiple plausible explanations to declining performance of vector control. To distinguish the population immunity effect observed in our model from waning vector control efficacy, a dengue control program would need to include on-going entomological and serological surveillance [49]. Because of the extreme epidemics we predict when abruptly stopping vector control, nearly five times pre-intervention years in the highest coverage scenario, distinguishing between these competing explanations is an important public health goal.

Entomological and epidemiological surveillance would inform the answer, but if those data are unclear or unavailable, ending a vector control program should be done gradually, to avoid potentially overwhelming the local healthcare system. We expect there are intervention scales more ambitious than those we analyzed—higher efficacy, higher coverage, longer durability, or more widespread application—where there would be little, if any, effectiveness waning. However, even these efforts would benefit from monitoring population immunity to understand the epidemic risk posed by rapid evolution of mosquito resistance [50].

While broad, active surveillance data is ideal, public health decision makers likely only have access to passive disease burden data. Via simulation, we have the luxury of comparing model worlds with and without interventions to determine intervention effectiveness, but the real world equivalent—randomized control trials—is impractical for large-scale interventions. Real world decisions must be based on the available disease burden data, which is an imperfect proxy for effectiveness. Burden can increase while both effectiveness and efficacy have been maintained, as other factors change like urbanization and climate. Our results for increasing overall force of infection (via increasing mosquito population) show relatively large increases in burden, but only moderate reduction in effectiveness for IRS-like interventions.

Though we specifically modeled dengue, the qualitative aspects of our results are useful for understanding dynamics of other similarly infectious, immunizing vector-borne diseases like chikungunya and Zika [51–53]. Because these viruses were novel in the Americas [54–56], there was no widespread population immunity to them, unlike what we see with dengue and which is required to obtain effectiveness in our model. Without pre-existing immunity, our model predicts very low effectiveness even with high vector control coverage. As such, achieving high effectiveness against emerging chikungunya and Zika with vector control likely would have required much more ambitious interventions than we consider here. As such, we should not have expected ongoing vector control efforts—short of eradication of *Ae. aegypti* and *Ae. albopictus*—to prevent those outbreaks. However, vector control targeting dengue will reduce cases of these and other *Aedes* transmitted arboviruses, especially in regions where these diseases have become endemic.

Our results indicate that IRS-like interventions are promising approaches to dengue control, but the ideal implementation of these interventions is location-specific. In addition to identifying optimal timing, intervention programs should incorporate a plan for continued monitoring to assess effectiveness and epidemic dynamics. Because of the high level of initial effectiveness in endemic regions, IRS might be combined with other approaches to accomplish local elimination, but new dengue introductions would have to be diligently prevented. The safest and most likely scenario to maintain long-term effectiveness is to combine a wide-spread vector control program with an efficacious vaccine to replace the natural immunization.

## Supporting information

S1 Text Further details on model assumptions and parameterization, and additional results. (PDF)
