# Supplementary material for: Forecasting the effectiveness of indoor residual spraying for reducing dengue burden
Source: PLoS Negl Trop Dis. 2018 Jun 25;12(6):e0006570. doi: 10.1371/journal.pntd.0006570 (PMC6042783; doi:10.1371/journal.pntd.0006570)
Supplement: S1 Text — (PDF) [file pntd.0006570.s001.pdf]

CONTENTS

	List of Figures	1
	List of Tables	1
S1	Updated dengue case data	2
S2	Model overview	3
	S2.1: Synthetic population model	3
	S2.2: Serotype of introductions	3
	S2.3: Factors affecting model seasonality	4
	S2.4: Mosquito initial infection age model	4
	S2.5: Mosquito lifespan model	5
	S2.6: IRS mortality model	5
	S2.7: IRS campaign model	6
	S2.8: Simulation eras	7
	S2.9: Effectiveness calculation	8
S3	Parameterization	8
	S3.1: Approximate Bayesian Computation (ABC) changes	8
	S3.2: Epidemic model parameter estimation	9
S4	Additional results	12
	References	20

LIST OF FIGURES

A	Factors affecting model seasonality.	4
B	Relationship between rollout period and instantaneous coverage	6
C	Impact of IRS on total number of mosquitoes	7
D	Shift in optimal timing due to alternate coverage and rollout	12
E	Shift in optimal timing due to alternate durability	13
F	Ten-year projections	14
G	Forty-year projections	15
H	Population immunity over time	16
I	Relative action of proactive vs reactive IRS	17
J	Effect of varying mosquito populations	18
K	Expanded main text Fig 1	19

LIST OF TABLES

A	Reported dengue cases from Yucatán, Mexico, 1979–2015	2
B	Fitted epidemic model parameters	10
C	Metrics used for epidemic model fitting	11

S1. UPDATED DENGUE CASE DATA

Year	Mild Cases	DHF/DSS Cases (%)	Total (per 100k)	Serotypes 1	2	3	4	Updated
1979	4234	0 (0.0)	4234 (409.8)	■	□	□	□	
1980	4672	0 (0.0)	4672 (439.2)	■	□	□	□	
1981	3377	0 (0.0)	3377 (308.8)	■	□	□	□	
1982	1412	0 (0.0)	1412 (125.7)	■	□	□	□	
1983	643	0 (0.0)	643 (55.7)	■	□	□	□	
1984	5486	9 (0.2)	5495 (464.3)	■	□	□	■	
1985	193	0 (0.0)	193 (15.9)	■	□	□	□	
1986	34	0 (0.0)	34 (2.7)	□	■	□	□	
1987	15	0 (0.0)	15 (1.2)	■	□	□	□	
1988	356	0 (0.0)	356 (27.3)	■	□	□	□	
1989	2	0 (0.0)	2 (0.2)	■	□	□	□	
1990	8	0 (0.0)	8 (0.6)	■	□	□	□	
1991	352	0 (0.0)	352 (25.1)	■	■	□	□	
1992	22	0 (0.0)	22 (1.5)	■	□	□	□	
1993	29	0 (0.0)	29 (2.0)	■	□	□	□	
1994	674	6 (0.9)	680 (44.8)	■	■	□	■	
1995	65	4 (5.8)	69 (4.4)	■	■	□	□	
1996	620	30 (4.6)	650 (41.2)	■	■	■	■	
1997	5366	163 (2.9)	5529 (346.2)	■	■	■	■	
1998	36	0 (0.0)	36 (2.2)	□	□	□	□	
1999	43	0 (0.0)	43 (2.6)	□	□	□	□	
2000	0	0 (NA)	0 (0.0)	□	□	□	□	
2001	252	36 (12.5)	288 (17.0)	□	■	■	□	†
2002	749	197 (20.8)	946 (54.9)	■	■	□	□	
2003	20	6 (23.1)	26 (1.5)	□	□	□	□	
2004	51	6 (10.5)	57 (3.2)	□	■	□	□	
2005	123	39 (24.1)	162 (8.9)	■	■	□	□	
2006	465	162 (25.8)	627 (34.0)	■	■	■	□	
2007	1472	390 (20.9)	1862 (99.4)	■	■	■	■	†
2008	573	149 (20.6)	722 (38.0)	■	■	□	□	†
2009	2109	1113 (34.5)	3222 (167.1)	■	■	□	□	†
2010	1706	817 (32.4)	2523 (129.0)	■	■	□	□	†
2011	4095	2124 (34.2)	6219 (313.6)	■	■	□	□	∗
2012	2899	2824 (49.3)	5723 (284.7)	■	■	■	■	∗
2013	1832	1032 (36.0)	2864 (140.6)	■	■	□	■	∗
2014	633	440 (41.0)	1073 (52.0)	■	■	□	■	⋄
2015	1129	376 (25.0)	1505 (71.9)	■	□	■	■	⋄

Table A. Reported dengue cases from Yucatán, Mexico, 1979–2015. ■ indicates serotype was reported, □ indicates not reported. Some data have been updated from [1, 2]: † indicates minor changes ($< 3\%$) in case counts, generally due to now including deaths in dengue hemorrhagic fever (DHF) and dengue shock syndrome (DSS) case counts; ∗ indicates revised (decreased) case counts, due to suspected cases previously treated as reported cases; ⋄ indicates new data reported in this publication.

S2. MODEL OVERVIEW

S2.1: Synthetic population model

This synthetic population model is similar to that described in [2]. The only change is how it represents aging.

As before, we model aging by transferring the complete disease and immune state from younger to older individuals once per year, which can be thought of as their birthday. For each person, the closest person who is one year younger "donates" their immune state on that birthday. Newborns (age 0 individuals) have no prior exposure, but may have maternal antibodies present contributed from a female in their household. As with the previous version of the model, individuals maintain the same age, gender, location, *et cetera* and only disease and immune states change.

In the previous version of the model, everyone in the model had the same birthday, namely day 99 of the Julian calendar, the approximate nadir of dengue transmission for Yucatán. This approach leads to a discontinuity in cases in the off-season, which was less relevant in [2] since there we no timing considerations; see [2], Fig. 2 and supplement section S2.3 for more details. In this work, however, we focus on timing questions, so we decided the model needed to eliminate that discontinuity.

In this version of the model, individuals have birthdays throughout the year. These birthdays are the same across simulation runs and across years within runs. Individuals' birthdays are uniformly distributed. We accomplish this by sorting individuals by age (oldest to youngest), and then using their index modulo 365 to determine what day of the year they will inherit disease and immune profiles.

S2.2: Serotype of introductions

The model generally introduces serotypes as in [2]. Like that work, before each simulation begins, we construct introduction time series independently for each of the four serotypes. For each serotype that is present, we sample a Poisson(λ_E) distribution for each day of the year to determine the number of exposures that should occur for that serotype, where $lambda_E$ is a fitted parameter.

For the fitting and historical periods, the model is identical to [2]. For the fitting period, we introduce the serotypes in the years in which they were observed. For historical periods with no recorded data, time series are generated for each of the four serotypes according to separately fit geometric distributions. In a given year there may be any combination of serotypes 1–4 introduced, including no serotypes. Each exposure applies to one randomly selected person in the population, who then becomes infected if susceptible.

However, serotype introduction for forecast (post-2015) years is handled differently. Because there has been a general trend toward hyperendemicity in Yucatán, including all four serotypes co-circulating in some years, we make the simplifying assumption that all serotypes will be introduced every year after 2015. In the previous work, the forecast period used geometric distributions like the historical period.

S2.3: Factors affecting model seasonality

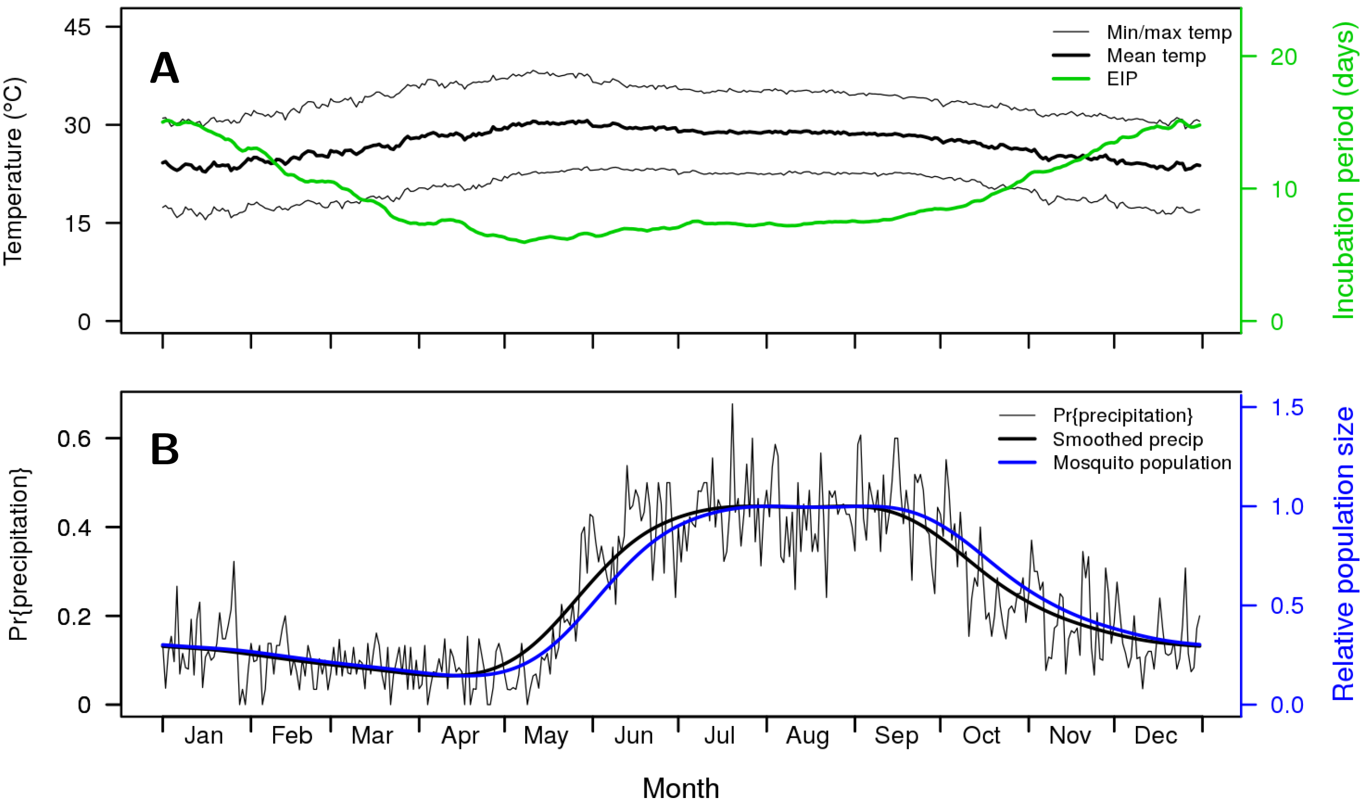

Figure A. Seasonality in the model is represented by a daily varying extrinsic incubation period (EIP; time between a mosquito biting and being able to transmit dengue) and mosquito population size. EIP is based on hourly temperature data, and mosquito population size is scaled based on historical frequency of rainfall for a given day. Climatological data are for Mérida, Mexico, 1979-2014; see S3.2 of [2] for details.

S2.4: Mosquito initial infection age model

In the previous version of the model, as a simplification we explicitly assumed that household infection history had no effect on mosquitoes age at initial infection. This means that we can always use the same model when selecting mosquito ages, but it neglects that mosquitoes will tend to be younger on their first bites in a household that has been infected awhile; older mosquitoes would likely have already been infected.

We have updated this assumption to instead assume that the current infection state in a household has, for the purpose of selecting a mosquitoes first infectious bite age, always been present. To ensure we were conservatively underestimating IRS benefit, we decided it was important to account for the greater likelihood of younger mosquitoes in infected households, since they would have reduced natural mortality due to senescence.

Specifically, the un-normalized probability that a mosquito is age a (in days) when it becomes infected is

$$Pr\{\text{Age} = a|\text{infecting bite}_{H\to M}\} \propto (1-\pi)^{a-1} * \pi * Pr\{\text{Age} = a\} \tag{1}$$

where $Pr\{\text{Age} = a\}$ is the age distribution of mosquitoes regardless of infection state, and π is the probability that a bite, weighted by time-of-day feeding preferences, would be infectious. In a household where no one leaves for work or school, π would simply be the fraction of people infectious. When a susceptible mosquito bites and becomes infected–and thus its age becomes important, since we need to know how long it will live and potentially spread dengue–we sample from Eq. 1 after normalization.

S2.5: Mosquito lifespan model

In the previous version of the model, once we drew a mosquitos initial biting age from a cumulative age distribution, we would then draw her death age from the same distribution by taking the remaining weight, re-normalizing it, and drawing on that new distribution.

However, the age distribution for a randomly selected member is not the same as the survival curve, though they are derivable from each other. For this particular distribution, the differences are also minimal and inconsequential to the overall qualitative results. However, with our changes to the mosquito infection age distribution, we have also switched to using the appropriate survival distribution.

S2.6: IRS mortality model

IRS has two effects on treated locations in the model: susceptible populations are reduced, and infectious mosquitoes are stochastically eliminated. We assume no effect on the introduction rate of mosquitoes at a location. We introduce IRS to houses as part of campaigns (see next section), and IRS affects houses for a fixed period from introduction: we focus on 90 day durability, but also consider 30 and 150 day durability. During that period, the IRS effects are constant and instantaneous–there is no ramp-up or waning.

Both effects are derived from assuming that IRS is 80% efficacious, $\epsilon_{\text{IRS}} = 0.8$, at reducing the mosquito population.

We represent the mosquito population reduction at location i by the fixed efficacy:

$$M_i^* = M_i(1 - \epsilon_{\text{IRS}}) \tag{2}$$

M_i is the untreated mosquito population and M_i^* is the treated population.

We assume this efficacy comes from a constant daily adult mortality probability, μ_{IRS}, on our normal mosquito population age distribution, though we do not modify that age distribution for the purpose of drawing infectious mosquitoes. We assume IRS has no impact on mosquito generation at a location. While there will no doubt be some effect, we do not have data to quantify that impact and we believe it is reasonable to assume it will not increase generation. Therefore, we are conservatively assuming there is no decrease. We expect there would be some decrease in generation, but since IRS does not act on the aquatic life stages stages (nor any mosquitoes activity outside households), and any local reduced egg-laying should be offset some by reduced larval competition, we anticipate similar emerging and immigrating populations. We can determine the daily mortality probability by solving the equations relating known parameters in terms of μ_{IRS}.

First, we know M_i in terms of the local mosquito generation (p_i, the age 0 mosquito population) and the model-wide conditional probability of mosquitoes dying on day j, μ_j:

$$M_i = p_i + p_i(1-\mu_1) + p_i(1-\mu_1)(1-\mu_2) + \ldots + p_i \prod_{k=1}^{n}(1-\mu_k) = p_i(1 + \sum_{j=1}^{n}\prod_{k=1}^{j}(1-\mu_k)) \tag{3}$$

We also know M_i* in similar terms, but with the additional daily IRS survival, $\sigma = 1 - \mu_{\text{IRS}}$.

$$M_i^* = p_i + p_i(1-\mu_1)\sigma + p_i(1-\mu_1)(1-\mu_2)\sigma^2 + \ldots + p_i\sigma^n \prod_{k=1}^{n}(1-\mu_k) = p_i(1 + \sum_{j=1}^{n}\sigma^j\prod_{k=1}^{j}(1-\mu_k)) \tag{4}$$

Since we know the relationship between M_i and M_i^*, we can combine these two equations and, using the GSL numerical solver `fsolver`, find μ_{IRS}. To reduce numerical error, we can use $\prod_{k=1}^{j}(1-\mu_k) = \frac{p_{i,j+1}}{p_{i,1}} = \hat{p}_{j+1}$, which we have from the CDF, and is identical for every location. This gives us:

$$-\epsilon_{\text{IRS}} + \sum_{j=1}^{n}(1 - \epsilon_{\text{IRS}} - \sigma^j)\hat{p}_{j+1} = 0 \tag{5}$$

For $\epsilon_{\text{IRS}} = 0.8$ and the daily mosquito mortality probabilities from our previous work, we obtain $1-\sigma = \mu_{\text{IRS}} = 0.13051$ or about 13% per day.

We apply this daily IRS mortality probability to infectious mosquitoes at treated locations, whether they originated there or migrated in, potentially causing them to die earlier than their lifespan drawn from the normal mosquito age distribution.

S2.7: IRS campaign model

People and mosquitoes in our model exist at, and move between, discrete locations in the categories houses (n = 376400), workplaces (n = 95560) and schools (n = 3402). We consider IRS campaigns that target houses at three different coverage (annual probability of treatment) levels, 25%, 50%, and 75%. We select houses independently for treatment for each year of the campaign, based on simulation run seed. Once selected for treatment in a given year, we randomly assign houses a treatment date within the campaign rollout period, which is either 1, 90, or 365 days. Treatment dates are drawn randomly with replacement, uniformly distributed throughout the rollout period. As with coverage, treatment start dates are redrawn randomly every year, and differ between runs.

For the main text and most supplemental results, we consider an IRS treatment that maintains efficacy (increasing mortality of local mosquitoes) for 90 days and then ceases to have an effect. In one sensitivity study, we considered alternate durabilities of 30 and 150 days; see results in SI Fig 4.1.

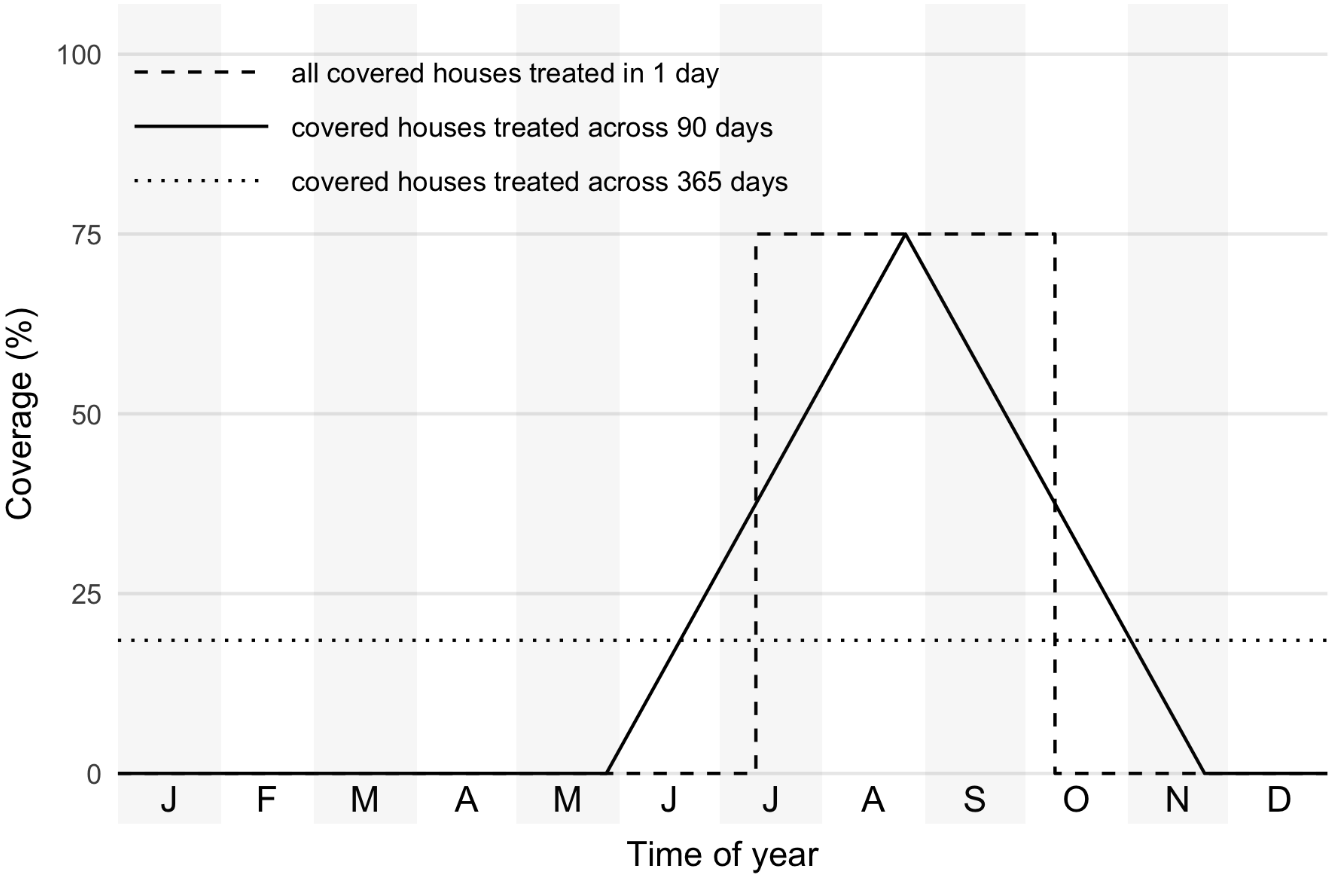

Figure B. Relationship between rollout period and expected instantaneous coverage, shown for treating 75% of households, with IRS that lasts 90 days, over the course of one year. 1-day and 90-day rollouts are shown using their approximate optimal start dates in mid July and late May, respectively. For earlier or later start dates, the curves would shift left or right, respectively. For coverage levels other than 75%, these curves would all rescale linearly, but their relative shapes and magnitudes would remain the same.

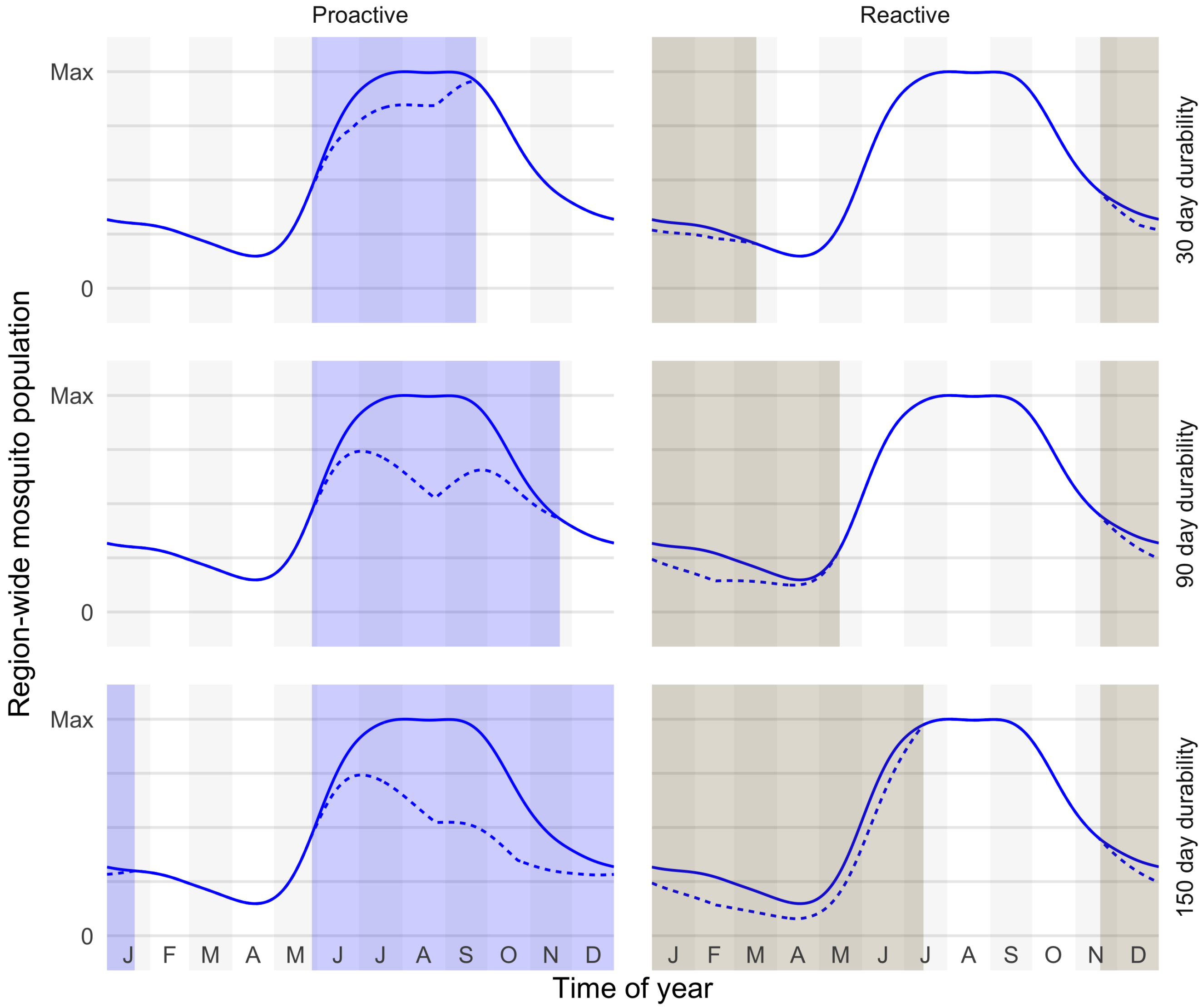

Figure C. Impact of IRS on total number of mosquitoes across all locations in the model. The solid lines (identical in each facet) show the baseline mosquito population. The dashed lines show the remaining mosquito population, relative to the baseline seasonal peak, for the different IRS timing and durability scenarios. All panels use 75% household coverage, 80% IRS efficacy within actively treated households, and 90 day campaign rollout. The facets are divided by proactive (blue) versus reactive (brown) campaigns, with the shaded region showing the total period of active insecticide in any household (*i.e.*, rollout duration plus durability). Some campaigns persist into the next calendar year, corresponding to the shaded regions extending from January.

S2.8: Simulation eras

As in [2], we represent historical eras: a burn-in period, a DDT era (with reduced mosquito populations), the fitting period (with particular serotype introductions) and a forecasting period. With the availability of new data, we have extended the fitting period to 2015. To study intervention effects on a system at approximate stationarity (*i.e.*, with epidemics that are not generally trending larger or smaller over time), we now include an additional stabilization period between the fitting era and forecasting. With one exception, for all results, this period lasts for 25 years, and uses the same serotype introduction model as the forecasting period. For force-of-infection-effect results (J), because we are changing the baseline number of mosquitoes in the population, we lengthen the stabilization period to 100 years to properly shift the population immunity and ensure stationarity.

S2.9: Effectiveness calculation

In previous work, we based effectiveness calculations on annual periods beginning on April 10 every year, consistent with when vaccination began in the model. Because we want to analyze the effect of IRS used at different times of the year, we cannot measure effectiveness from the same date for all scenarios. Using a single date can misleadingly penalize interventions with start times far away from a set date by including extra cases before the intervention started, or ending aggregation of cases before the effects of the intervention had ended. Instead we measure cases in "intervention years"—*i.e.*, all the cases that occur in a year from the first date of the intervention. However, to minimize computation, baseline annual incidence is calculated using January 1; the same incidence would be observed for any annual period because the model is at approximate stationarity.

S3. PARAMETERIZATION

Assumed parameters have not changed from [2], including incubation and infectious periods, and parameters governing movement, immunity, and mosquito biting. Fitting the remaining parameters is described in the sections below.

S3.1: Approximate Bayesian Computation (ABC) changes

Parameter estimation is performed using AbcSmc as described in [2] with the following changes:

1.
   - Previous: Parameters for all sets were sampled from independent (marginal) distributions.
   - Current: Parameters for the first set are still sampled from independent priors, but in subsequent sets we sample from a joint distribution, namely a multivariate Gaussian distribution fitted to the particles selected from the most recent SMC set. Where parameters are correlated, this leverages covariance information from the previous best particles to propose parameter combinations that are more likely to yield simulated metrics that fit observed metrics. If parameters are not correlated, this change has no effect.

2.
   - Previous: To reduce computation time, we first fit a Yucatán subpopulation corresponding to the city of Mérida (approximately half the population of Yucatán) for seven SMC sets, then used that posterior as the prior for fitting the full Yucatán population for seven more sets. All sets used $N = 10000$ particles with accepted fraction $\rho = 0.01$. The posterior was estimated from the best 100 particles from the seven Yucatán sets.
   - Current: We now improve fitting efficiency using a different approach. We observed during past fitting procedures that estimates for model parameters change dramatically during early SMC sets, and only subtly during later sets. This shift is likely because each new particle represents a relatively large increase in information about how the model responds to parameter changes. Later SMC sets are better-informed and thus benefit from more thorough sampling to continue refining the posterior.

     In this work we therefore use smaller initial SMC sets, followed by larger sets: $N = 1000$, 2500, 5000, and 5000 particles for sets 1-4, 10000 particles each for sets 5-10. All SMC sets use the full Yucatán population, and the same number of particles is accepted from each set (*i.e.* the accepted fraction ρ is adjusted such that $\rho N = 500$). The posterior is estimated from the best 1000 particles across all ten sets.

In other regards, the fitting procedure remains the same as in [2]. Pseudocode of our ABC-SMC algorithm, updated to reflect the above changes, is as follows, where N is the number of parameter combinations sampled, ρ is the fraction retained in the predictive prior/posterior, and $n = \lfloor \rho * N \rfloor$ is the size of the predictive prior/posterior:

1. Set the SMC iterator $t = 1$.
   (a) For sample iterator $i = 1, \ldots, N$:
      i. Sample parameter vector $\theta_i^{(1)}$ independently from prior distributions $\pi(\theta)$
      ii. Simulate data ("metrics") $x_i^{(1)} \sim p(x_i^{(1)} | \theta_i^{(1)})$.
2. Calculate PLS model for $\theta^{(t)}$ and $x^{(t)}$
3. Transform observed data and each $x_i^{(t)}$ to new independent, orthogonal metrics using PLS model

4. Calculate Euclidean distance between transformed observed and transformed simulated metrics
5. Set $\Theta^{(t)}$ = best $\theta^{(t)}$, the best n samples, ranked by smallest distance.
6. If $t > 1$ and Θ has converged, stop here. Otherwise:
   (a) Set ζ_{t+1} equal to the variance-covariance of $\Theta^{(t)}$
   (b) Update diagonal (variance) values of ζ_{t+1} to be twice the variance of $\Theta^{(t)}$
   (c) For $j = 1, ..., n$:
      i. Set weight $\omega_j^{(t)} \propto \begin{cases} 1/n & : t = 1 \\ \pi(\Theta_j^{(t)}) / \sum_{k=1}^{n} \omega_j^{(t-1)} K(\Theta_j^{(t)} | \Theta_k^{(t-1)}; \zeta_t) & : t > 1 \end{cases}$
      where $K(\Theta_j^{(t)} | \Theta_k^{(t-1)}; \zeta_t)$ is a multivariate Gaussian perturbation kernel with mean $\Theta_k^{(t-1)}$ and variance-covariance ζ_t, evaluated at $\Theta_j^{(t)}$.
   (d) Normalize $\omega_i^{(t)}$ to sum to 1.
   (e) Set $t = t + 1$
      i. For $i = 1, \ldots, N$:
         A. Choose θ_i^* from $\Theta^{(t-1)}$ with probabilities $\omega^{(t-1)}$.
         B. Sample $\theta_i^{(t)}$ from $MultivariateGaussian(\theta_i^*, \zeta_t)$
         C. Simulate data $x_i^{(t)} \sim p(x_i^{(t)} | \theta_i^{(t)})$.
   (f) Go to step 2.

S3.2: Epidemic model parameter estimation

The remaining model parameters, described in Table B, were estimated using the ABC-SMC procedure described above to identify parameter combinations (Θ) that produce dynamics similar to the observed data. For "similarity", we consider the following 21 metrics (x):

- descriptive statistics for annual reported cases per 100,000 people, 1979–2015
  - mean, all quartiles, standard deviation, skewness, annual autocorrelation (median crossing rate)
- seroprevalence in Mérida children aged 8–14, 1987
- pre-1995 severe proportion: fraction of reported cases that were severe, 1979–1994
- modern severe proportion: fraction of reported cases that were severe, 1995–2015
- seroprevalence in Mérida residents, 2014, stratified by age class (9 metrics)

The mean, quartiles, standard deviation, and skewness have the conventional definitions. A median crossing is defined as a sequential pair of years with one year above and one below the whole series median, and the median crossing rate is the number of median crossings per total years considered minus one.

Available serosurvey data include two 1987 surveys of school children aged 8–14, both within the municipality of Mérida, found to have seroprevalences of 56.3% and 63.7% [3]. We do not know how representative these samples were of the population of all 8–14 year-olds in Mérida, but they are nonetheless valuable in identifying the infection:reported case ratio. We assume an empirical seroprevalence of 60% for 8–14 year-olds in Mérida in 1987. When the simulator reaches April 9, 1987, we tally the seroprevalence for that subgroup of the Yucatán population as one of the metrics.

Observed values for metrics and their posterior distributions from the model are described in Table C.

The parameters fitted to the metrics are:

- RF_m, reported fraction of mild (but still symptomatic) cases
- $UR_{m,95}$, pre-1995 under-reporting of mild cases, *e.g.* ratio of pre-1995 reported fraction to 1995 and later reported fraction
- RF_s, reported fraction of severe cases

- SP, secondary pathogenicity: fraction of secondary infections that are cases
- SS, secondary severity: fraction of secondary cases that are severe
- $PSSR$, primary severity:secondary severity ratio
- λ_E, daily mean exposure introduction rate
- M_{peak}, seasonal peak of average mosquito population size per location

Priors for these parameters are described in Table B.

Parameter	Sampled Prior	Modeled Prior		Posterior	
		Median	Range	Median	95% IR
RF_m	$\mathcal{N}$(-2.944439, 1.4)	0.0595	$[0.01, 1]$	0.0179	$[0.0100, 0.155]$
$UR_{m,95}$	$\mathcal{N}$(-0.5,1)	0.0406	$[0.01, 1]$	0.0112	$[0.0100, 0.0535]$
RF_s	$\mathcal{N}$(-1.098612, 0.8)	0.265	$[0.02, 1]$	0.250	$[0.0321, 0.891]$
SP	$\mathcal{N}$(0.8472979, 0.7)	0.7	$[0, 1]$	0.557	$[0.172, 0.954]$
SS	$\mathcal{N}$(-2.197225, 2.25)	0.1	$[0, 1]$	0.0265	$[0.000131, 0.275]$
$PSSR$	$\mathcal{N}$(0.0, 1.0)	0.5	$[0, 1]$	0.363	$[0.0229, 0.883]$
λ_E	$\mathcal{N}$(-4.5, 1.5)	0.275	$[0, 25]$	0.0189	$[0.00183, 12.9]$
M_{peak}	$\mathcal{N}$(0, 0.7)	60.5	$[1, 120]$	46.4	$[29.7, 117]$

Table B. Prior distributions (sampled and modeled) and posterior medians and 95% interquantile ranges (IR) for fitted epidemic model parameters. Logistic transformations were used to convert sampled parameter values into model parameter values based on the ranges specified. RF_m is the reported fraction for mild disease; $UR_{m,95}$ is the fraction by which mild cases are under-reported pre-1995; RF_s is the reported fraction for severe disease; SP is the reference (secondary infection with DENV 1) pathogenicity; SS is reference (secondary case) probability of severe disease; $PSSR$ is the relative-risk of severe disease in primary cases; λ_E is the introduction rate (number of exposures per serotype per day, for the entire modeled population); M_{peak} is the mean number of mosquitoes per location, on the day when mosquito populations are largest. See Section S3 for further details.

Metric	Observed	Posterior		
		Mean	Median	95% IR
mean	101	115	99.7	$[26.2, 286]$
minimum	0.00	0.0399	0.00	$[0.00, 0.502]$
25th percentile	2.73	4.59	0.190	$[0.00, 25.2]$
median	38.0	22.5	7.73	$[0.000551, 88.2]$
75th percentile	129	116	98.0	$[5.05, 337]$
maximum	464	757	632	$[167, 2030]$
stdev	140	199	166	$[43.9, 532]$
skewness	1.38	1.95	2.00	$[1.35, 2.44]$
median-crossing rate	0.250	0.258	0.250	$[0.167, 0.333]$
1987 seroprevalence, 8–14 yo	0.600	0.540	0.573	$[0.154, 0.873]$
pre-1995 severe proportion	0.000 697	0.0801	0.0744	$[0.0224, 0.174]$
modern severe proportion	0.290	0.135	0.123	$[0.0434, 0.275]$
2014 seroprevalence, 0–4 yo	0.278	0.218	0.231	$[0.00471, 0.468]$
2014 seroprevalence, 5–9 yo	0.490	0.474	0.466	$[0.227, 0.777]$
2014 seroprevalence, 10–14 yo	0.616	0.676	0.648	$[0.457, 0.944]$
2014 seroprevalence, 15–19 yo	0.727	0.778	0.760	$[0.577, 0.984]$
2014 seroprevalence, 20–29 yo	0.741	0.853	0.848	$[0.657, 0.996]$
2014 seroprevalence, 30–39 yo	0.708	0.939	0.945	$[0.808, 1.00]$
2014 seroprevalence, 40–49 yo	0.808	0.971	0.981	$[0.878, 1.00]$
2014 seroprevalence, 50–59 yo	0.742	0.986	0.994	$[0.913, 1.00]$
2014 seroprevalence, 60+ yo	0.828	0.995	0.999	$[0.958, 1.00]$

Table C. Observed values and posterior means and medians for epidemic model metrics. Posterior values are for simulations on the Yucatán population after averaging across 10 realizations for each of the 100 parameter combinations that resulted from the fitting procedure. Seroprevalence is for 8–14 year old Mérida residents (subset from the simulated Yucatán population) on April 9, 1987. IQR is interquantile range.

S4. ADDITIONAL RESULTS

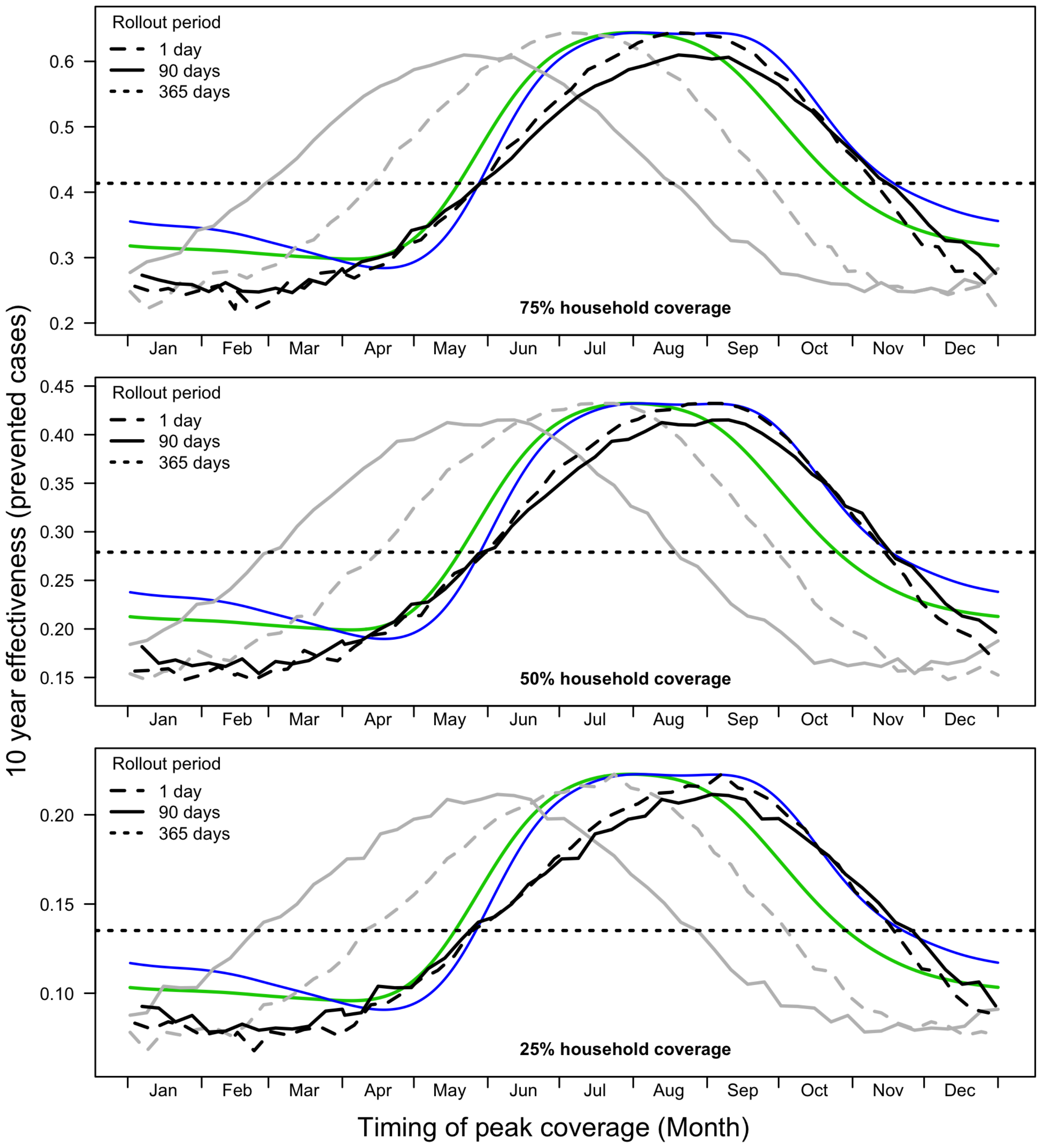

Figure D. Effect of timing of peak coverage on IRS campaign effectiveness for the rollout periods and coverage levels we considered. R_0 (green) and mosquito population (blue) seasonality are rescaled and shown here to allow for comparison with the effectiveness curves (black). The scale is adjusted for each coverage level to highlight trends. Across all coverage levels, optimally-timed 1-day rollouts slightly out-performed 90-day rollouts but had start dates later in the year, values for effectiveness minima were insensitive to rollout period, and continuous campaigns performed worse than optimally timed campaigns, but better than the worst timings.

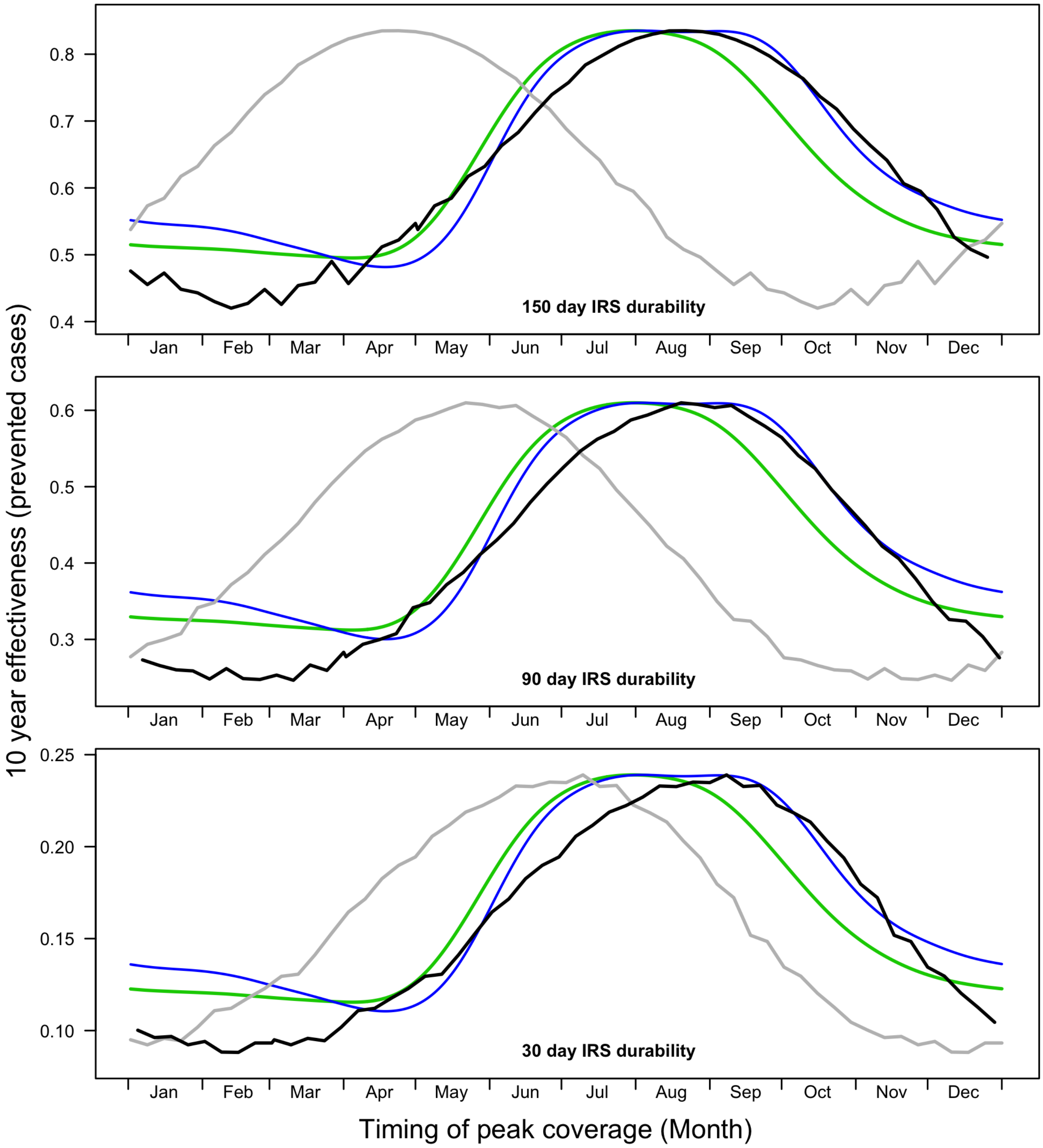

Figure E. Effect of timing of peak coverage on IRS campaign effectiveness for the insecticide durabilities (how long the insecticide continues to kill mosquitoes after application) we considered. R_0 (green) and mosquito population (blue) seasonality are rescaled and shown here to allow for comparison with the effectiveness curves (black). The scale is adjusted for each durability to highlight trends.

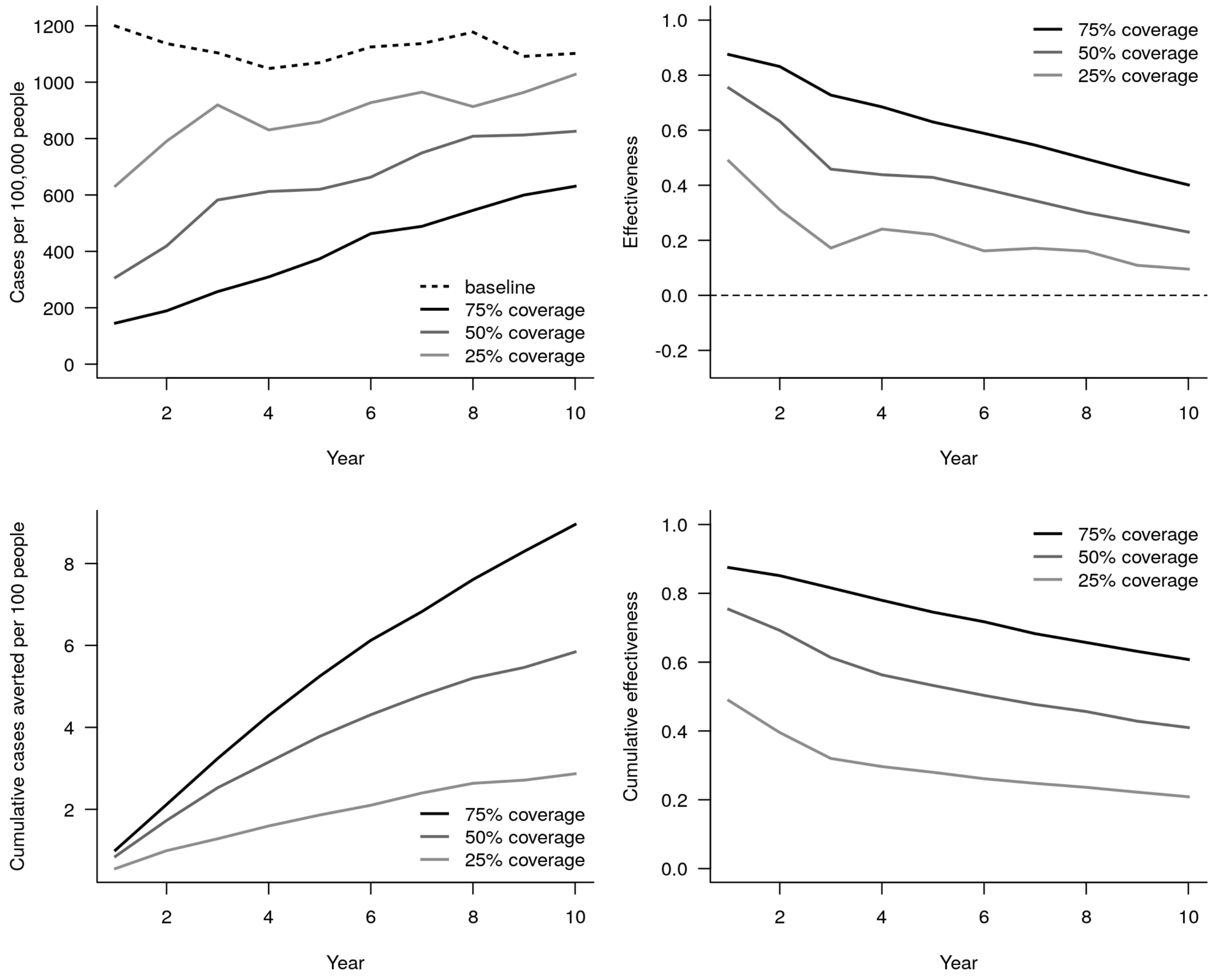

Figure F. Ten-year projections. The underlying reason for reduced effectiveness can clearly be attributed to gradually increasing case rates after introduction of the intervention.

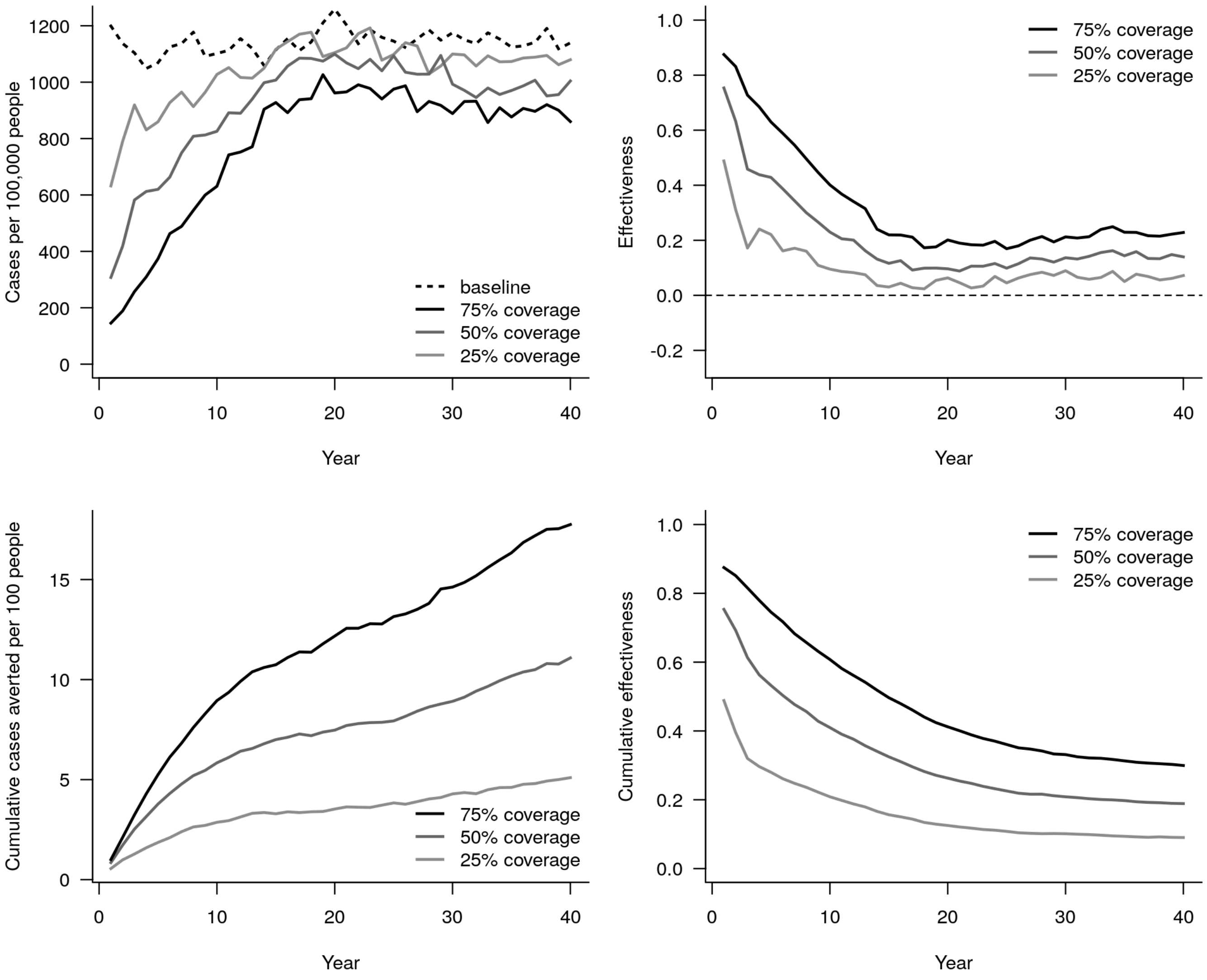

Figure G. Forty-year projections. The steady-state dynamics become fairly clear starting around year 30.

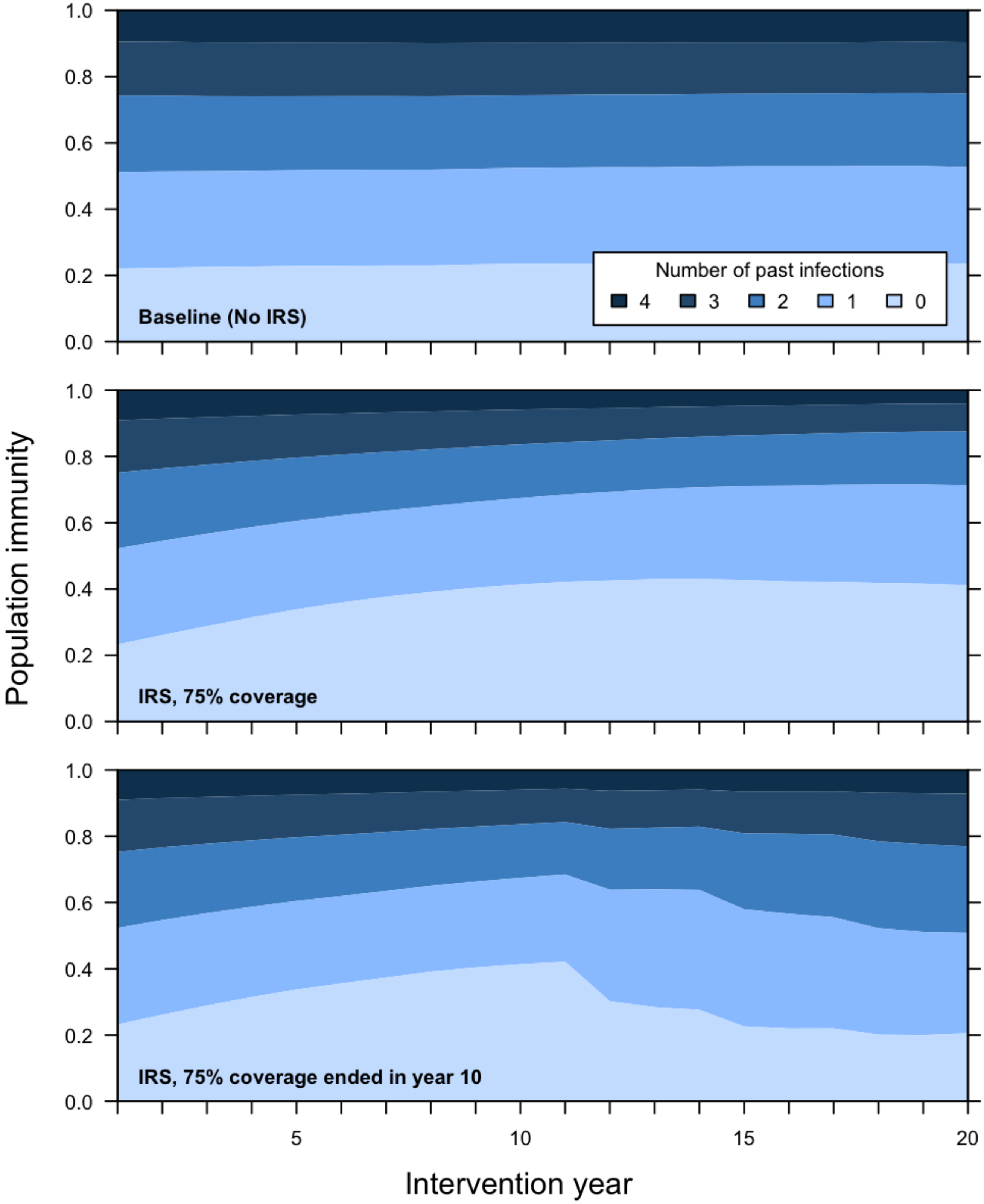

Figure H. Population immunity over time. The top panel shows the stable population seroprevalence. The second panel shows the declining infection history in the population. The final panel shows what occurs when the intervention is abruptly stopped; note that population-level seroprevalence has returned to roughly pre-intervention levels by year 20.

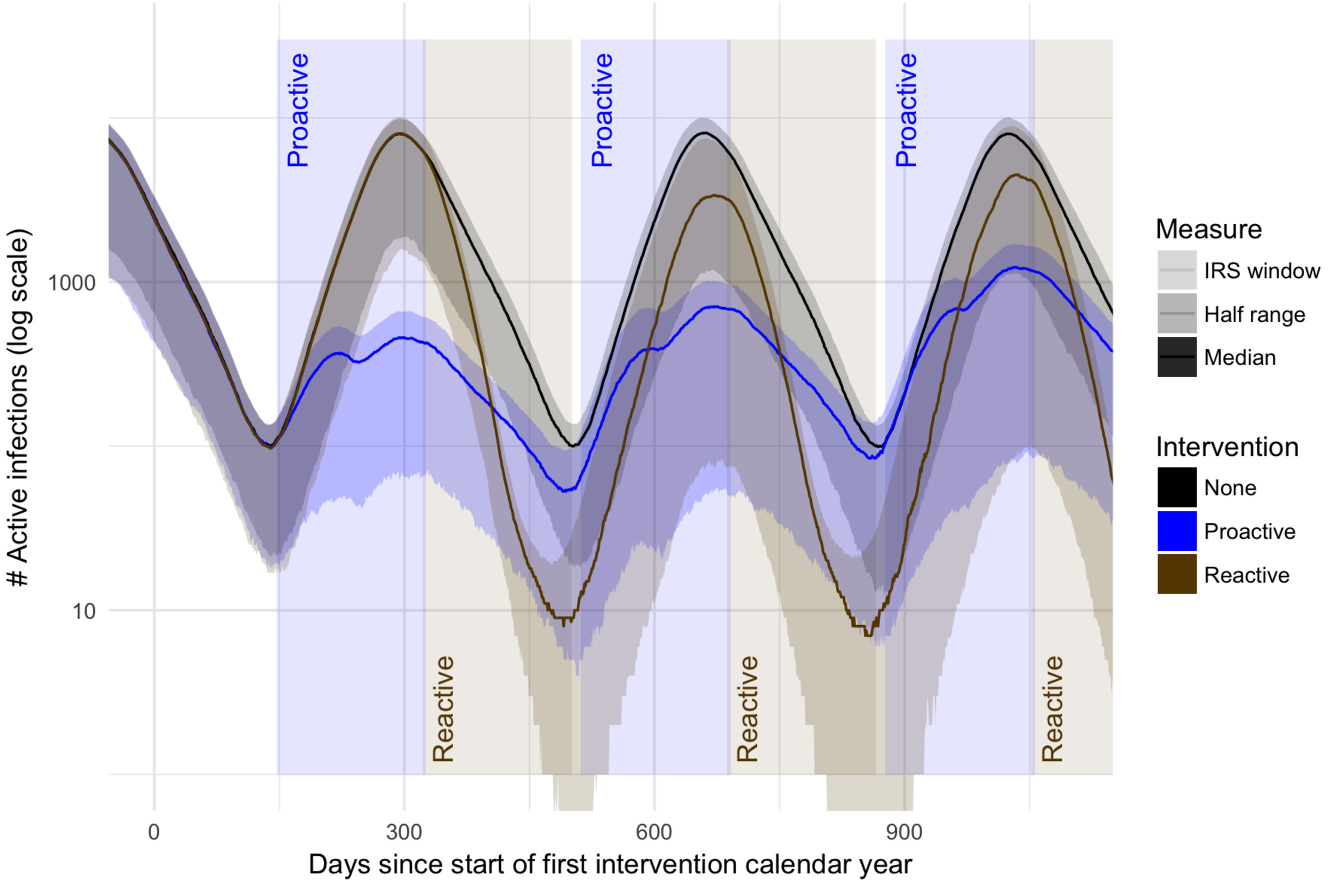

Figure I. Relative action of proactive vs reactive IRS. Proactive IRS dampens a normal epidemic by reducing the mosquito population. The small second lump corresponds to IRS durability beginning to wear off in houses treated early in the campaign, before seasonal changes turn the epidemic. Reactive IRS instead sharply curtails the epidemic at the end of the season, minimizing the inter-seasonal transmission and disrupting any low-probability transmission chains, but has no direct effect during the epidemic. Reactive IRS tends to ensure minimal active infections at the start of the high-transmission season, delaying the epidemic by a few doubling periods.

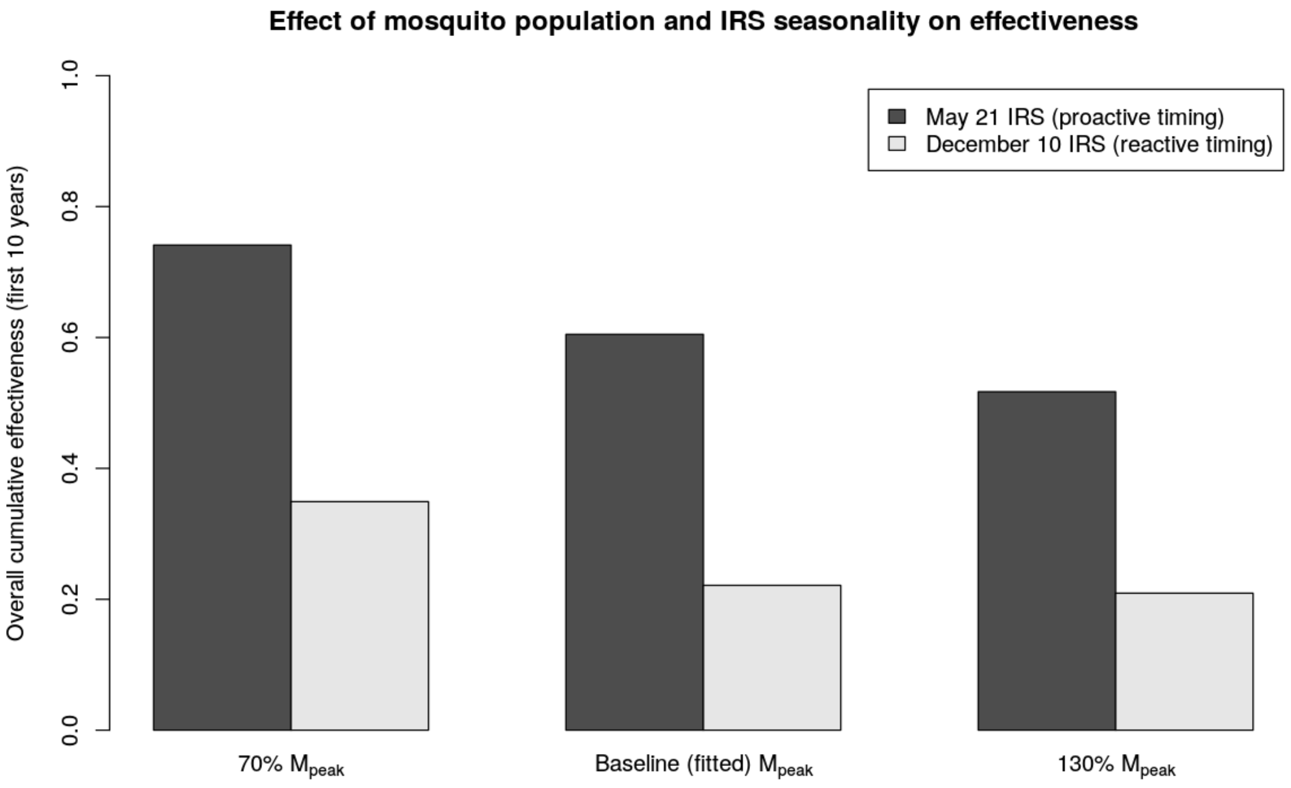

Figure J. Effect of varying mosquito populations. These results and those in the main text highlight some important dynamic trends, which can be understood by considering limiting extremes in mosquito population. At an extreme high number of mosquitoes, even a substantial reduction via high coverage and efficacy still leaves enough mosquitoes transmit dengue to most of the susceptible population. At the other extreme, if there were just barely enough mosquitoes to sustain an outbreak, any reductions to the population would prevent outbreaks entirely, leaving only imported cases and very short transmission chains. So, regions with higher mosquito populations should see diminished effectiveness, even for the same efficacy and coverage levels, while lower mosquito density regions would see higher effectiveness.

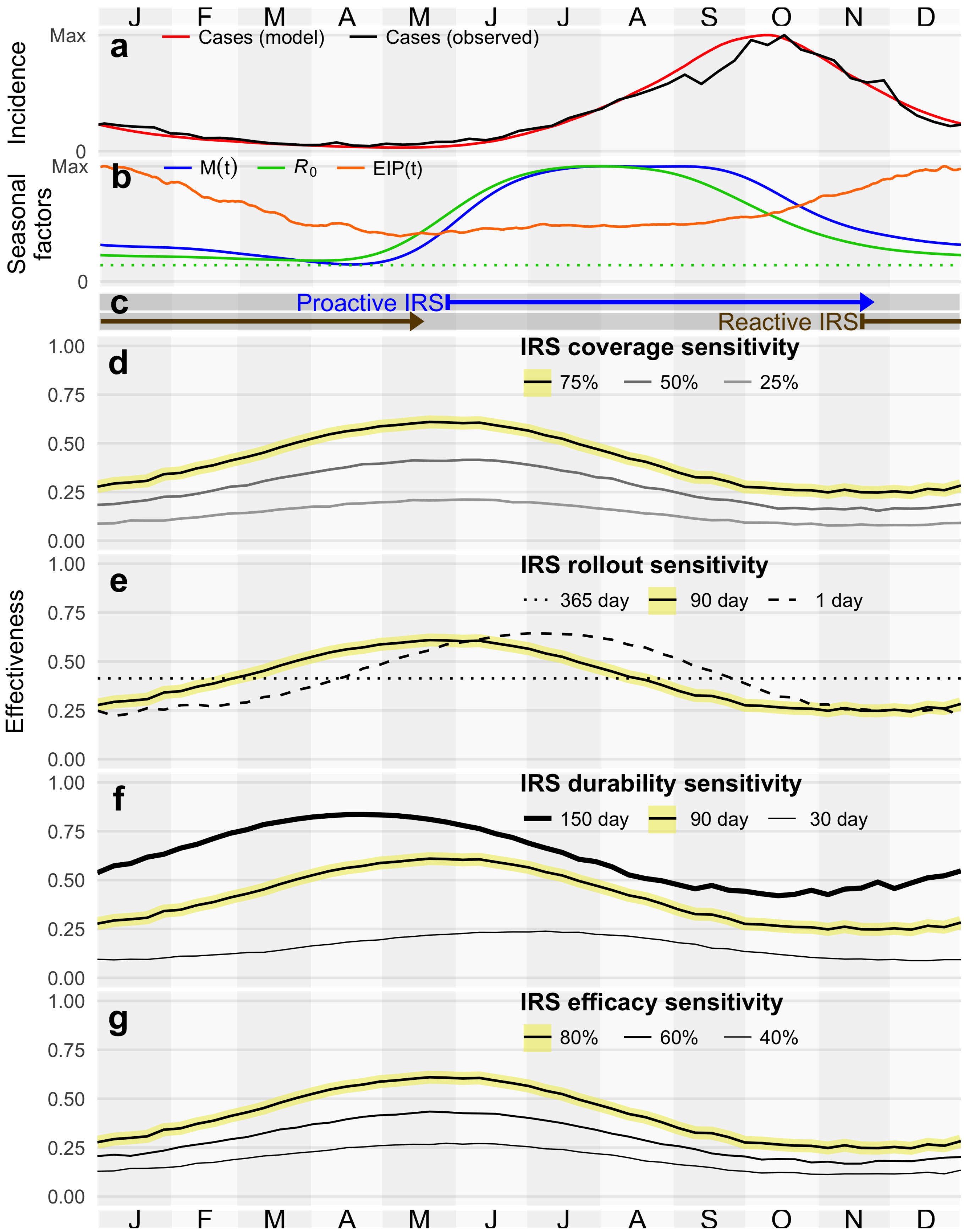

Figure K. Expanded main text Fig 1. Panel (g) shows the detail results for the IRS efficacy study, which is qualitatively similar to the sensitivity to coverage. The other panels are reproduced here for context.

[1] Dirección General de Epidemiología, SINAVE, Ministry of Health, Mexico, "Dengue morbidity report," (2015).
[2] T. J. Hladish, C. A. Pearson, D. L. Chao, D. P. Rojas, G. L. Recchia, H. Gómez-Dantés, M. E. Halloran, J. R. Pulliam, and I. M. Longini, PLoS Negl Trop Dis **10**, e0004661 (2016).
[3] J. Farfan-Ale and M. Lorono-Pino, Boletin Medico del Hospital Infantil de Mexico **48**, 780 (1991).
